# Adverse effects following anti–COVID-19 vaccination with mRNA-based BNT162b2 are alleviated by altering the route of administration and correlate with baseline enrichment of T and NK cell genes

**DOI:** 10.1371/journal.pbio.3001643

**Published:** 2022-05-31

**Authors:** Ayesa Syenina, Esther S. Gan, Justin Z. N. Toh, Ruklanthi de Alwis, Lowell Z. Lin, Christine Y. L. Tham, Jia Xin Yee, Yan Shan Leong, Huizhen Sam, Charlene Cheong, Yii Ean Teh, Ian L. E. Wee, Dorothy H. L. Ng, Kuan Rong Chan, Jean X. Y. Sim, Shirin Kalimuddin, Eugenia Z. Ong, Jenny G. Low, Eng Eong Ooi

**Affiliations:** 1 Program in Emerging Infectious Diseases, Duke-NUS Medical School, Singapore; 2 Viral Research and Experimental Medicine Centre, SingHealth Duke-NUS Academic Medical Centre, Singapore; 3 School of Life Sciences, Nanyang Polytechnic, Singapore; 4 Department of Infectious Diseases, Singapore General Hospital, Singapore; 5 Saw Swee Hock School of Public Health, National University of Singapore, Singapore; The University of Texas Medical Branch at Galveston, UNITED STATES

## Abstract

Ensuring high vaccination and even booster vaccination coverage is critical in preventing severe Coronavirus Disease 2019 (COVID-19). Among the various COVID-19 vaccines currently in use, the mRNA vaccines have shown remarkable effectiveness. However, systemic adverse events (AEs), such as postvaccination fatigue, are prevalent following mRNA vaccination, and the underpinnings of which are not understood. Herein, we found that higher baseline expression of genes related to T and NK cell exhaustion and suppression were positively correlated with the development of moderately severe fatigue after Pfizer-BioNTech BNT162b2 vaccination; increased expression of genes associated with T and NK cell exhaustion and suppression reacted to vaccination were associated with greater levels of innate immune activation at 1 day postvaccination. We further found, in a mouse model, that altering the route of vaccination from intramuscular (i.m.) to subcutaneous (s.c.) could lessen the pro-inflammatory response and correspondingly the extent of systemic AEs; the humoral immune response to BNT162b2 vaccination was not compromised. Instead, it is possible that the s.c. route could improve cytotoxic CD8 T-cell responses to BNT162b2 vaccination. Our findings thus provide a glimpse of the molecular basis of postvaccination fatigue from mRNA vaccination and suggest a readily translatable solution to minimize systemic AEs.

## Abstract 2

Systemic adverse events, such as post-vaccination fatigue, are prevalent consequences of mRNA vaccination; why is this? This study shows that higher baseline expression of T and NK cell genes increases susceptibility to fatigue after mRNA vaccination, and that altering the route of vaccination may reduce the incidence of mRNA vaccine-associated systemic adverse events.

## Introduction

The Severe Acute Respiratory Syndrome Coronavirus 2 (SARS-CoV-2) has caused 100s of millions cases of Coronavirus Disease 2019 (COVID-19) globally. Through airborne and droplet transmission, SARS-CoV-2 has spread rapidly to cause a pandemic [1,2] and genetic variants of SARS-CoV-2, such as Delta and Omicron, have and will continue to emerge to worsen and prolong the pandemic. Fortunately, timely development and deployment of COVID-19 vaccines have begun to reduce the impact of COVID-19 on global health and economies. Among these vaccines, the most efficacious ones have been the mRNA-based vaccines, namely BNT162b2 and mRNA-1273 developed by Pfizer-BioNTech and Moderna, respectively. However, while these vaccines are remarkably efficacious against COVID-19, they are reactogenic [3,4]. Over 50% of people who have received either of these vaccines have reported systemic adverse events (AEs) [5]. These symptoms, especially fatigue, are self-limiting, although they can be debilitating [3,5–8]. Such systemic AEs are thought to contribute, at least to some extent, to the hesitancy to be vaccinated or to be boosted after completion of the primary 2-dose vaccination series [9–12].

We have previously shown genomic-level host susceptibility factors to systemic AEs [13]. Using the live-attenuated yellow fever vaccine, we found increased endoplasmic reticulum stress and reduced tricarboxylic acid cycle activities at prevaccination baseline to be associated systemic AEs [13]; such baseline states were associated with increased early innate immune and pro-inflammatory responses to vaccination that were correlated with systemic AEs [14]. It is thus possible that systemic AEs that develop following mRNA vaccination are not stochastic events but rather are shaped by host susceptibility factors. Moreover, if indeed a propensity to inflammation underpins systemic AEs, then it may be possible to reduce the rate and severity of systemic AEs by altering the route of vaccination [15,16]. Indeed, it has previously been shown that despite eliciting similar immunogenicity, intramuscular (i.m.) inoculation produced more systemic AEs than subcutaneous (s.c.) inoculation [15]. Both the currently deployed mRNA vaccines as well as those undergoing clinical trials have used i.m. inoculation as the route of vaccination; how alternative routes of vaccination impact mRNA vaccine immunogenicity and reactogenicity has not been systematically explored.

In this study, we examined the molecular determinants of fatigue, a commonly reported systemic AE following vaccination with BNT162b2, in a cohort of healthcare workers. Using whole blood transcriptomic profiling, we found that higher baseline expression of genes related to exhaustion and suppression of T and NK cell activities was associated with the development of moderately severe fatigue. Furthermore, baseline expression of these T and NK cell genes was positively correlated with expression of immune activating genes at day 1 postvaccination. We also found, using a mouse model, that the pro-inflammatory and complement response to BNT162b2 vaccination could be minimized by altering the route of vaccination from i.m. to s.c.) inoculation. Importantly, neither vaccine immunogenicity nor efficacy was compromised by altering the route of vaccination.

## Results

### Characteristics of participants and AEs

**Fig 1:**
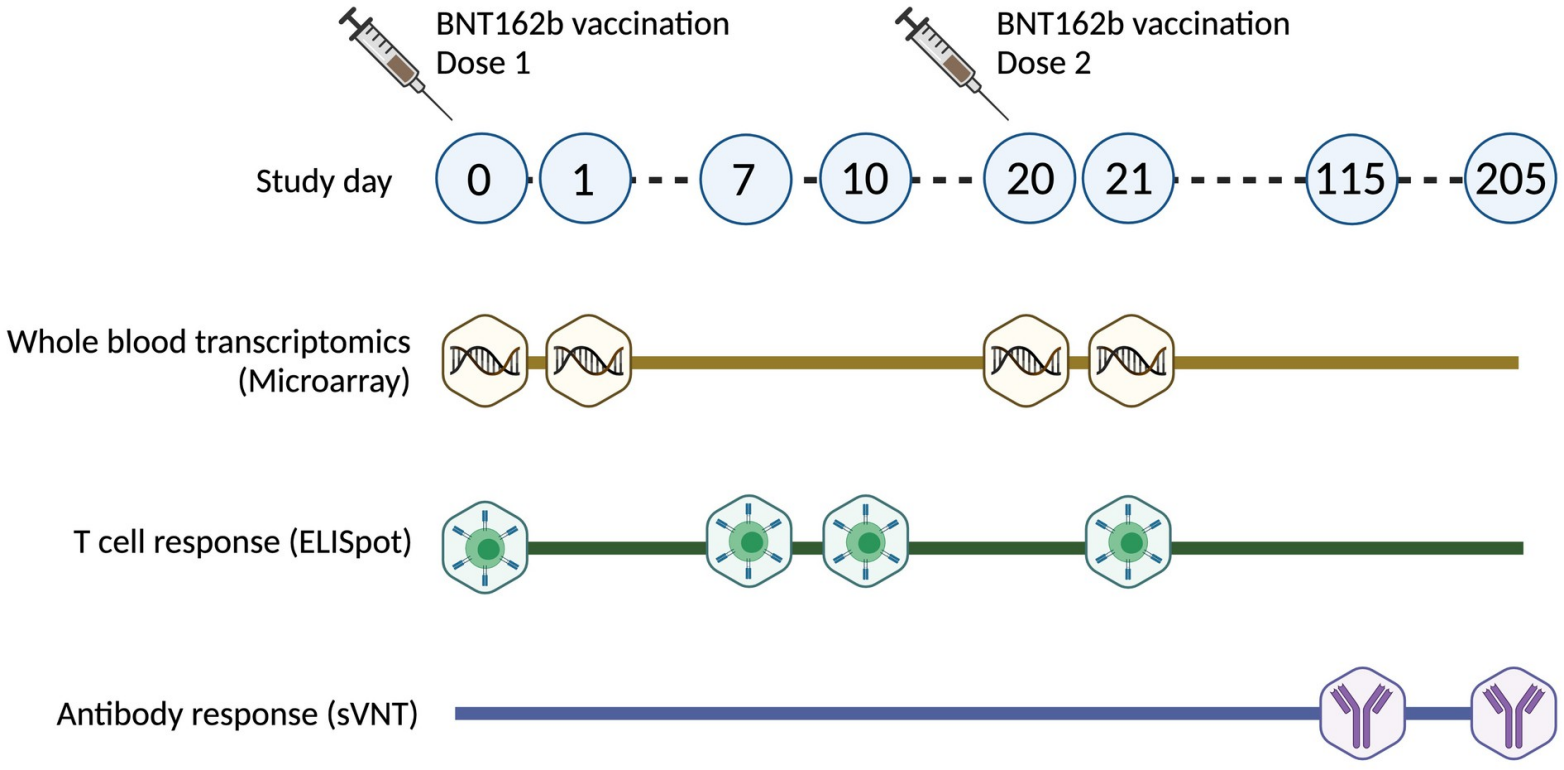
Overview of study design. Timeline of dose 1 and dose 2 vaccination and sample collection for gene expression studies, T-cell, and antibody response analysis. sVNT, surrogate virus neutralization test.

**Fig 2:**
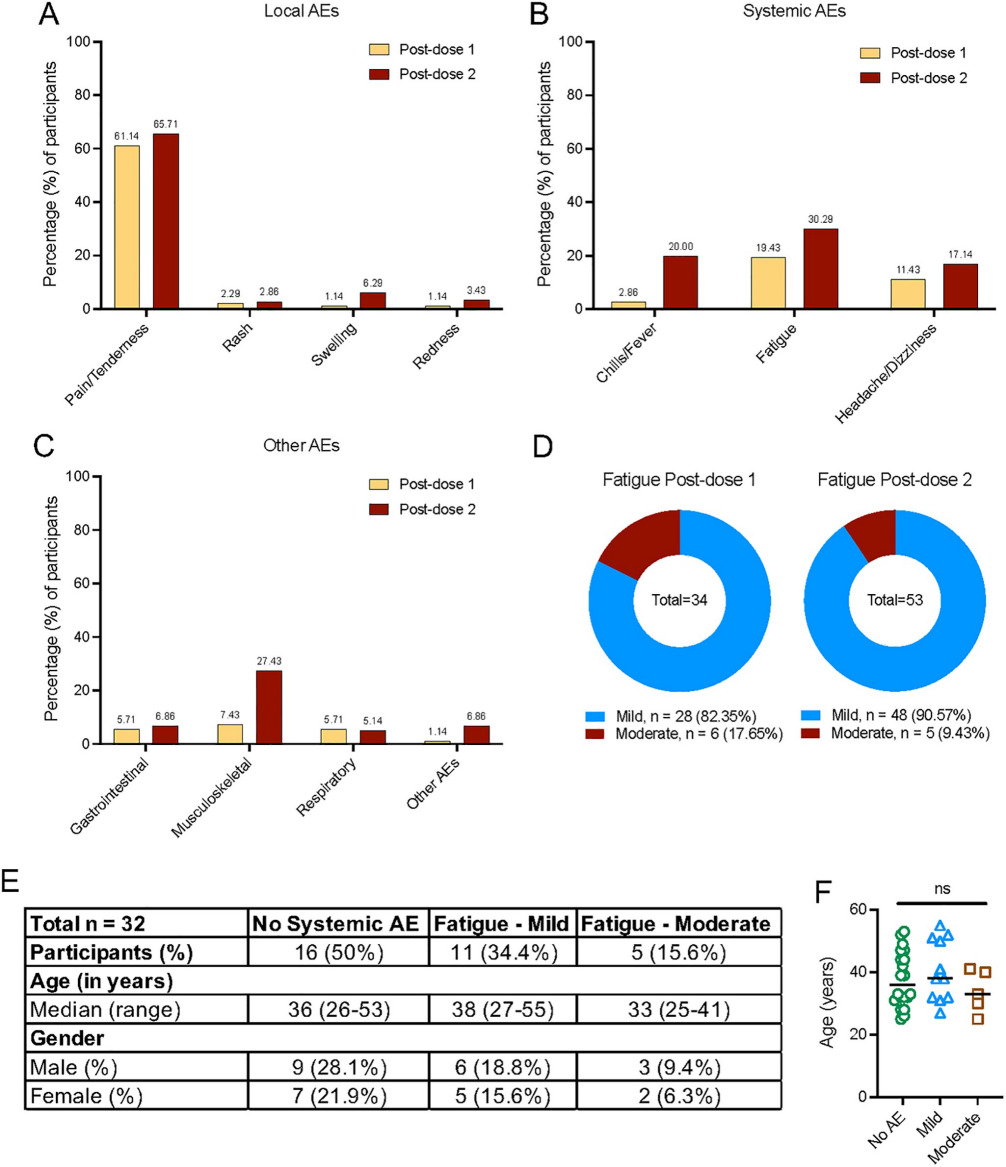
Reported local and systemic AEs following vaccination with the Pfizer-BioNTech (BNT162b2) vaccine (*n* = 175). **(A)** Percentage of participants reporting local AEs at site of injection following dose 1 and 2 of vaccination. **(B)** Percentage of participants reporting systemic AEs following dose 1 and 2 of vaccination. **(C)** Percentage of participants reporting AEs associated with respiratory, gastrointestinal, musculoskeletal and other reported AEs that does not fall under any categories. **(D)** Percentage of participants reporting fatigue categorized by severity (mild and moderately severe) following dose 1 and 2 of vaccination. **(E)** Demographics of participants selected in the nested case control study. **(F)** Age of participants categorized based on fatigue severity. Data underlying this figure can be found in S1 Data. AE, adverse event.

An overview of the study is shown in Fig 1. A total of 175 healthcare workers who received the Pfizer-BioNTech (BNT162b2) COVID-19 vaccine were enrolled to our study. Demographics of participants included in this study are shown in S1 Table. Participants were monitored for onset of AEs within the first 7 days of receiving dose 1 and dose 2 of the vaccine. Participants who reported AEs were followed up until resolution of AEs. We classified AEs by system organ class according to the Common Terminology Criteria for Adverse Events (CTCAE) version 4.0. Local AEs (Fig 2A) included the development of pain or tenderness, rash, swelling, and redness at the site of injection, while systemic AEs (Fig 2B) included fever, chills, and fatigue. AEs were also subcategorized into gastrointestinal (abdominal pain, diarrhea, and nausea), musculoskeletal (arthralgia and myalgia), central nervous system (headache and dizziness), and respiratory (cough, sore throat, and runny nose) (Fig 2C). Other reported AEs included palpitations, cervical lymphadenopathy, exacerbated hay fever symptoms, heightened sense of smell, malaise, and swelling of lymph nodes (Fig 2C).

More than half of the study participants experienced local AEs, namely pain and tenderness, postdose 1 (61.14%) and postdose 2 (65.71%) (Fig 2A). The most common systemic AE was fatigue, similar to observations reported elsewhere [3,5–8]. In our cohort, 19.43% (34/175) and 30.29% (53/175) participants reported fatigue postdose 1 and 2, respectively (Fig 2B). Of these, 17.65% and 9.43% experienced moderately severe fatigue after dose 1 and 2, respectively (Fig 2D). We defined, a priori, mild fatigue as fatigue with no interference with activity and moderately severe fatigue as fatigue with some interference with activity (S2 Table) [17].

### Moderately severe fatigue was associated with T and NK cell–related genes at baseline

To determine if there were baseline host susceptibility factors for fatigue following mRNA vaccination, we conducted a nested case control study within our prospectively enrolled cohort. Using convenience sampling, we included 16 participants who reported fatigue following vaccination [mild fatigue (*n* = 11) and moderately severe fatigue (*n* = 5)], where sufficient blood was available for RNA extraction, and compared these against age- and gender-matched controls (*n* = 16) who did not report any AEs postvaccination (Fig 2E and 2F). Whole blood samples were collected on study days 0 (predose 1), 1, 20 (predose 2), and 21. Total RNA was extracted from whole blood and subjected to microarray profiling for gene expression studies (Fig 1).

**Fig 3:**
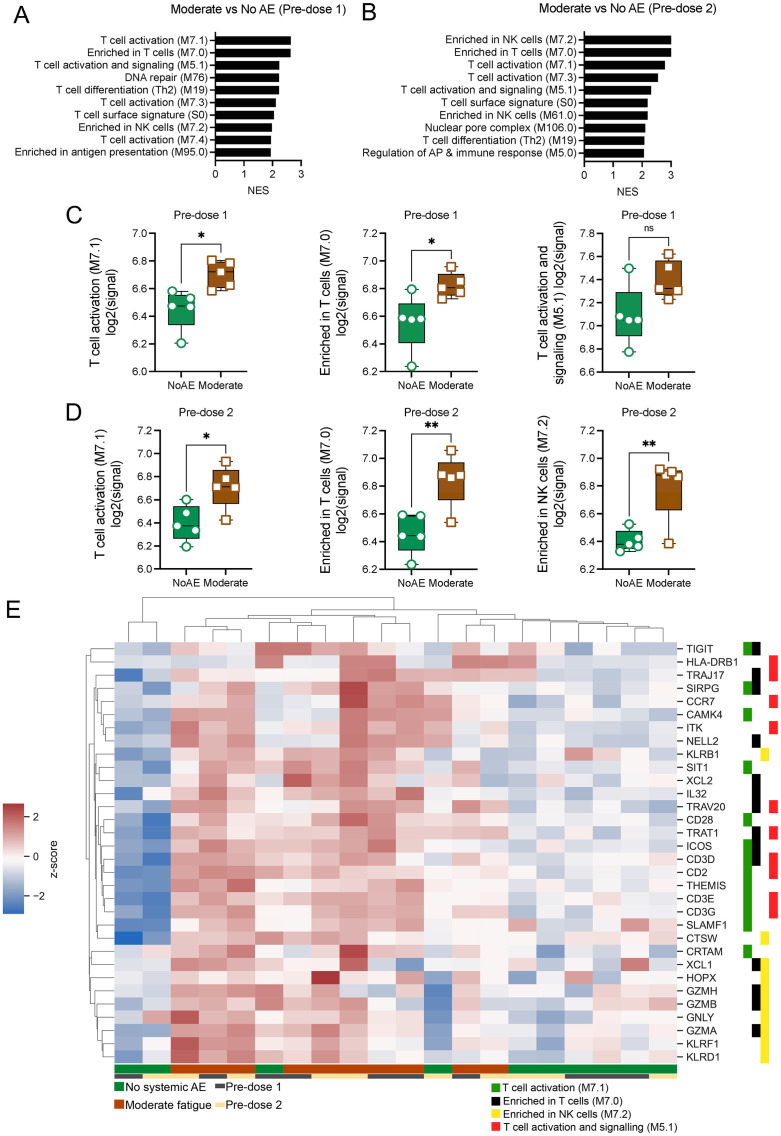
GSEA on whole genome transcripts from participants with moderately severe fatigue and their age- and gender-matched controls reveal differences in baseline expression of genes in T and NK cell–related pathways. Direct comparisons between moderately severe fatigue versus no systemic AE were made. Genes were preranked according to their log2FC for GSEA analysis using the BTM. **(A, B)** Top 10 positively enriched pathways in participants with moderately severe fatigue compared to no systemic AE at predose 1 (A) and predose 2 (B). NES values are displayed. FDR <0.05 cutoff values were imposed. **(C, D)** Mean log2 signal of all genes in the top enriched pathways at predose 1 (C) and predose 2 (D), categorized by participants with either moderately severe fatigue or no systemic AE. **(E)** Gene expression of the top 10 LEGs in the top enriched pathways at predose 1 and 2. Z scores of raw log2 signal are displayed. Data presented as means ± SD. Unpaired Student *t* test were used for experiments comparing 2 groups. *P < 0.05, **P < 0.01, ****P < 0.0001. *n* = 5 for each group. Data underlying this figure can be found in S1 Data. AE, adverse event; BTM, blood transcription module; FDR, false discovery rate; GSEA, gene set enrichment analysis; LEG, leading edge gene; NES, normalized enrichment score.

Gene set enrichment analysis (GSEA) on preranked gene lists was next conducted using previously established blood transcription modules (BTMs) [18]. Genes were ranked by fold change (FC) in cases relative to controls at either predose 1 or predose 2. This analysis identified positive enrichment of gene sets related to T cells, as well as those related to NK cells and antigen presentation in participants with moderately severe fatigue compared to no AE controls at both predose 1 and predose 2 (Fig 3A and 3B). Baseline expression of genes in the top 3 pathways identified at both these time points were either significantly higher or tended to trend higher in those with moderately severe fatigue compared to controls (Fig 3C and 3D). Unsupervised clustering of the top 10 leading edge genes (LEGs) from each of the identified pathways further underscored their association with moderately severe fatigue, with lower baseline expression of these genes observed in participants with no systemic AEs (Fig 3E). Notably, among the top genes identified were genes previously implicated in T and NK cell exhaustion such as T-cell immunoglobulin and ITIM domain (TIGIT) [19] and killer cell lectin-like receptor F1 (KLRF1). Genes known to have an inhibitory effect on T and NK cell cytotoxicity, including TIGIT [20], KLRF1 [21], and KLRD1 [22,23], were also increased in participants with moderately severe fatigue as compared to controls.

**Fig 4:**
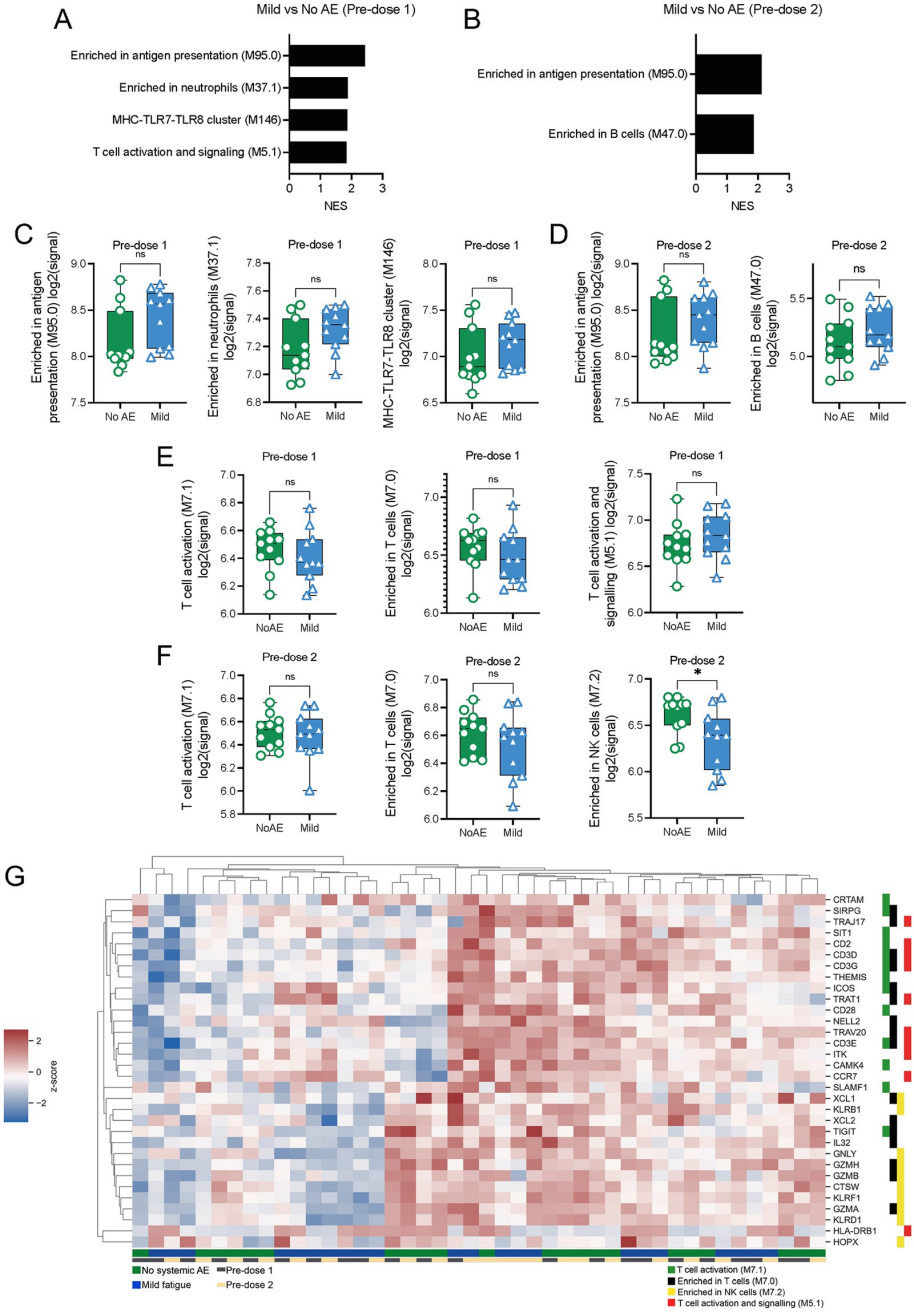
GSEA on whole genome transcripts from participants with mild fatigue showed few differences at baseline compared to their age- and gender-matched controls. Direct comparisons between mild fatigue versus no systemic AEs were made. Genes were preranked according to their log2FC for GSEA analysis using the BTM. **(A, B)** Top positively enriched pathways in participants with mild fatigue compared to no systemic AE at predose 1 (A) and predose 2 (B). NES values are displayed. FDR <0.05 cutoff values were imposed. **(C, D)** Mean log2 signal of all genes in the top enriched pathways at predose 1 (C) and predose 2 (D), categorized by participants with either mild fatigue or no systemic AE. **(E, F)** Mean log2 signal of all genes in the top enriched pathways previously identified in moderately severe participants at predose 1 (E) and predose 2 (F), categorized by participants with either mild fatigue or no systemic AE. **(G)** Gene expression of the top 10 LEGs in the top enriched pathways at predose 1 and 2. Z scores of raw log2 signal are displayed. Data presented as means ± SD. Unpaired Student *t* test were used for experiments comparing 2 groups. *P < 0.05, **P < 0.01, ****P < 0.0001. *n* = 11 for each group. Data underlying this figure can be found in S1 Data. AE, adverse event; BTM, blood transcription module; FDR, false discovery rate; GSEA, gene set enrichment analysis; LEG, leading edge gene; NES, normalized enrichment score.

Unlike the comparison between cases with moderately severe fatigue and their respective age- and gender-matched controls, fewer pathways were positively enriched in participants with mild fatigue compared to their respective controls at both predose 1 and predose 2 baselines (Fig 4A–4D). Unlike what was observed in participants with moderately severe fatigue, the top pathways enriched in participants with mild fatigue were genes enriched in antigen presentation, neutrophils, and B cells (Fig 4A–4D). However, the difference in baseline expression of genes in these pathways between those with mild fatigue compared to their matched controls did not reach statistical significance, at either predose 1 (Fig 4C) or predose 2 (Fig 4D). Likewise, with the exception of genes associated with NK cells at predose 2, there were no significant differences in baseline expression of T-cell–related genes between mild fatigue cases compared to controls (Fig 4E and 4F). Unsupervised clustering of the top 10 genes identified in these pathways also failed to clearly differentiate cases with mild fatigue from controls (Fig 4G).

We confirmed our whole genome microarray findings using the Nanostring nCounter platform to quantify mRNA transcripts (S1A–S1G Fig). Consistently, genes associated with T and NK cells were increased in participants with moderately severe fatigue compared to those with mild fatigue or controls (S1A–S1G Fig). These findings collectively suggest that individuals with increased basal level of transcripts associated with T and NK cell activity and function may be more prone to developing fatigue with a greater degree of severity after BNT162b2 vaccination.

### Immune genes postvaccination are positively correlated with elevated expression of T-cell genes at baseline

We next investigated how these baseline gene expression differences influenced the transcriptomic changes postvaccination. To do this, we performed GSEA by using genes ranked by correlation between baseline expression (average log2signal for all genes represented in indicated gene sets) of positively enriched pathways and baseline-normalized log2 FC of gene expression at day 1 postvaccination relative to baseline expression.

**Fig 5:**
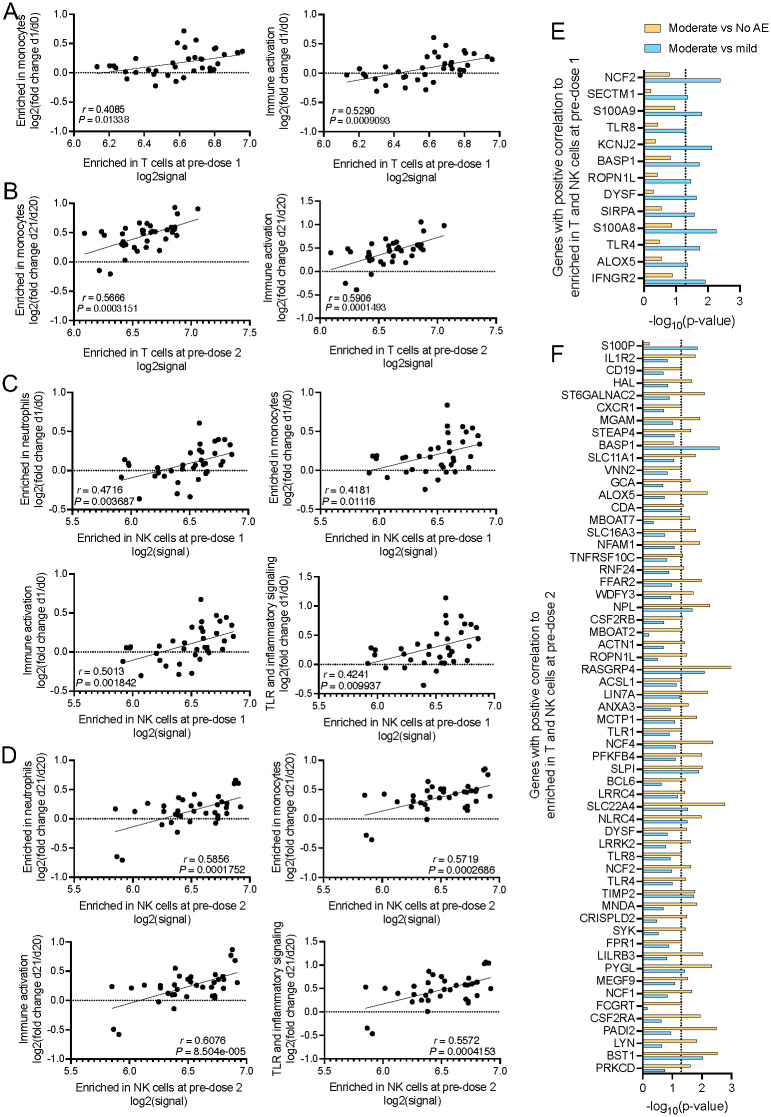
Baseline expression of T and NK cell associated genes were positively correlated with induction of immune genes at day 1 postvaccination. GSEA (FDR < 0.05) was performed to identify enriched gene sets in gene lists where genes were ranked according to their correlation between baseline expression of positively enriched pathways and baseline-normalized log_2_ FC of genes at 1 day postvaccination. Total *n* = 32 participants. **(A, B)** Correlation for baseline expression of genes from the enriched in T-cells gene set and LEGs from the enriched in monocytes or immune activation gene sets at 1 day postdose 1 (A) or postdose 2 (B). **(C, D)** Correlation for baseline expression of genes from the enriched in NK cells gene set and LEGs from the enriched in neutrophils, enriched in monocytes, immune activation, and TLR and inflammatory signaling gene sets at 1 day postdose 1 (C) or postdose 2 (D). **(E, F)** *P* values from unpaired Student *t* test for LEGs in genes in all enriched pathways at 1 day postdose 1 (E) or postdose 2 (F). Only genes that are expressed at higher levels in moderately severe fatigue participants relative to those with mild fatigue and no systemic AEs are shown. Data underlying this figure can be found in S1 Data. AE, adverse event; FC, fold change; FDR, false discovery rate; GSEA, gene set enrichment analysis; LEG, leading edge gene; TLR, Toll-like receptor.

From our GSEA analysis, we selected the top pathways that were enriched at both dose 1 and dose 2. We found that the changes in expression of genes in monocyte and immune activation pathways were positively correlated with the level of gene expression in the enriched in T-cells pathway at its respective predose 1 and predose 2 baseline (Fig 5A and 5B). Similarly, the level of expression of genes associated with monocytes, neutrophils, immune activation, and Toll-like receptor (TLR) and inflammatory signaling at day 1 following first and second dose of vaccination were positively correlated with the baseline expression of genes in the enriched in NK cells pathway (Fig 5C and 5D). Moreover, elevated baseline expression of KLRF1, which stimulates cytotoxicity and cytokine release in NK cells [24], was positively correlated with expression of genes enriched in monocytes and genes associated with cell cycle and transcription at 1 day postvaccination (S2A and S2B Fig).

We next examined if the genes represented in the above pathways were differentially expressed in participants with moderately severe fatigue, relative to those with mild fatigue or no systemic AE (Fig 5E and 5F, S2C and S2D Fig). Notably, IFNGR2, TLR8, TLR4, TLR1, and CSF2RB were among the genes found to be expressed at significantly higher levels in participants with moderately severe fatigue (Fig 5E and 5F, S2C and S2D Fig). These same genes were previously found to be associated with AEs following live yellow fever vaccination [14]. Additionally, we observed that there were markedly more genes identified postdose 2 compared to postdose 1 (Fig 5E and 5F). This is congruent with previous studies that found more stimulation of immune genes were found after second dose of vaccination compared to the first dose [25]. These findings suggest that a more robust activation of innate immune genes on day 1 postvaccination could contribute to the development of fatigue post-BNT162b2 vaccination.

The association between upregulation of T and NK cell related genes at baseline with moderately severe fatigue raises the possibility that vaccine-induced immune responses may be affected by such systemic AE. However, we observed no statistically significant difference in both antibody and T-cell responses postvaccination, as measured by the surrogate virus neutralization test (sVNT) [26] and interferon gamma (IFNγ) ELISpot, respectively, among participants that developed either moderately severe fatigue, mild fatigue, and those with no systemic AEs (S2E and S2F Fig).

### Reduced levels of reactogenicity is observed with inoculation via the s.c. route compared to i.m. route, in vivo

While our findings indicate correlation of baseline genes enriched in T and NK cells with postvaccination fatigue, additional in vivo experimentation will be needed to demonstrate their functional role in this systemic AE, if any. However, even if a functional role could be quickly established, it is unlikely that the current mRNA vaccines can be immediately redesigned or reformulated to minimize postvaccination fatigue and other systemic AEs. We thus took a more pragmatic line of investigation to explore if modifying the route of administration of the BNT162b2 mRNA vaccine from i.m. to s.c. could reduce systemic reactogenicity.

The design of the in vivo study in a K18 human angiotensin converting enzyme 2 (K18-hACE2) transgenic mouse model [27,28], is shown in S3 Fig. Two groups of K18-hACE2 mice (*n* = 5 per group) were vaccinated with 5 μg (50 μL) of BNT162b2 via the s.c. (inoculation site was the loose skin over the neck) and i.m. (inoculation site was the right rectus femoris) routes, respectively. Two control groups (*n* = 5 per group) received 50 μL PBS via similar routes. This vaccine dose was chosen as it was the maximum volume that could be delivered i.m. in mice (S3 Fig). Weights and clinical scores were also monitored in all 5 mice for 10 days postvaccination (S3 Table).

**Fig 6:**
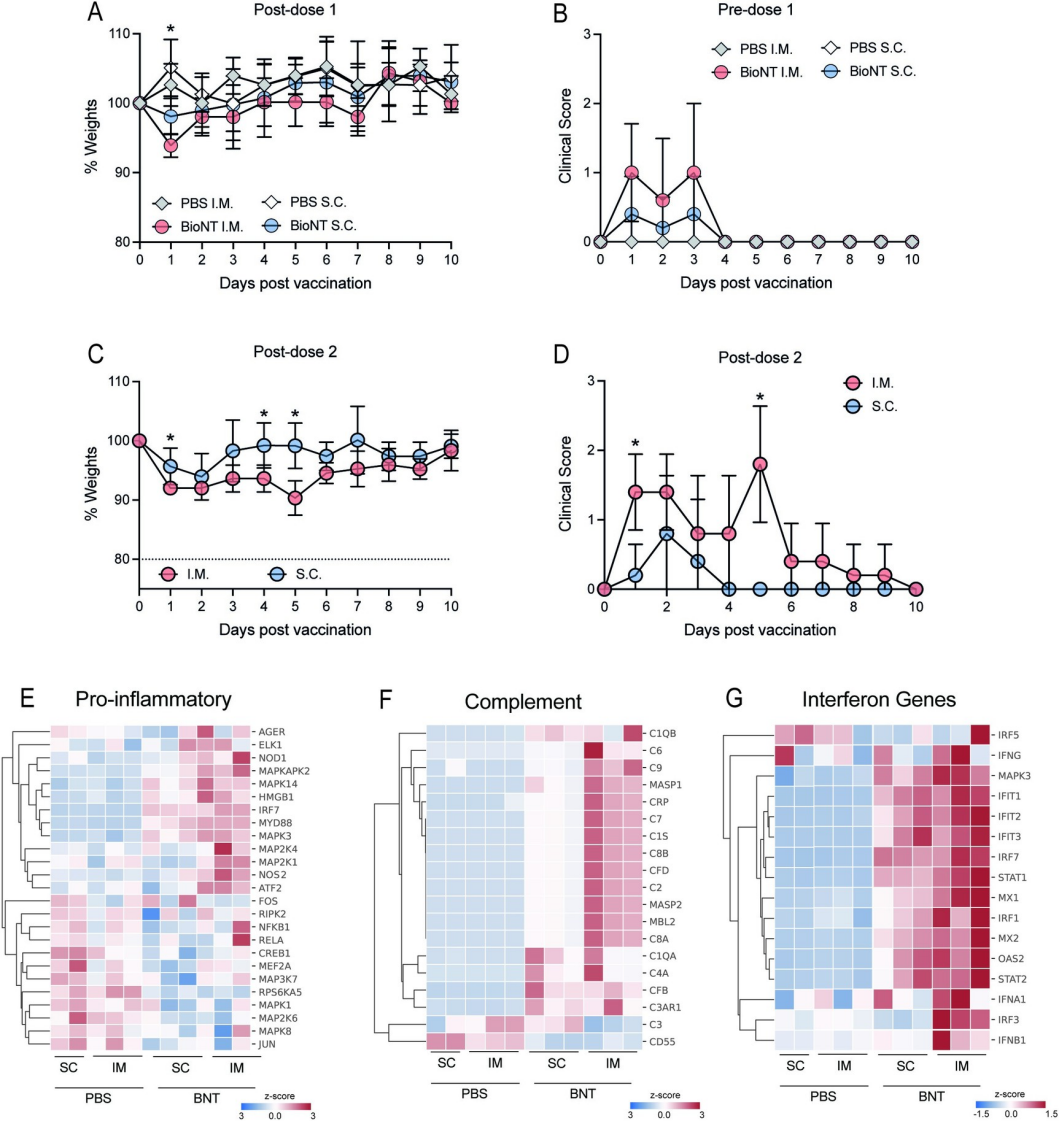
BNT162b2 vaccination via the s.c. route resulted in reduced weight loss, clinical scores and inflammation compared to vaccination via the conventional i.m. route. **(A)** Weights of animals assessed for the first 10 days postdose 1. **(B)** Clinical scores of animals assessed for the first 10 days postdose 1. **(C)** Weights of animals assessed for the first 10 days postdose 2. **(D)** Clinical scores of animals assessed for the first 10 days postdose 2. **(E–G)** Expression of pro-inflammatory (E), complement (F) and IFN response (G) genes in whole blood presented as heatmap of z-scores. Data presented as means ± SD. In vivo experiments were conducted with a minimum of *n* = 5 per group, except for gene expression study with *n* = 3 per group. Unpaired Student *t* test was used for experiments comparing 2 groups. *P < 0.05. Data underlying this figure can be found in S1 Data. IFN, interferon; i.m., intramuscular; s.c., subcutaneous.

At postdose 1, animals that received i.m. BNT162b2 displayed greater weight loss compared to those that received s.c. BNT162b2 (Fig 6A); no weight loss was observed in those that received either i.m. or s.c. PBS control. This weight loss, however, was only seen at day 1 postvaccination, and all other clinical scores were comparable between the 2 groups (Fig 6A and 6B). At postdose 2, animals that received s.c. BNT162b2 showed weight loss compared to their control animals that received s.c. PBS. However, the extent and duration of weight loss were significantly reduced compared those observed in animals that received i.m. BNT162b2 (Fig 6C). Moreover, clinical scores in animals that received i.m. BNT162b2 were significantly higher than those that received s.c. BNT162b2 (Fig 6D).

In a separate group of mice (*n* = 3) each, we examined the effect of s.c. compared to i.m. inoculation on innate immune gene expression in whole blood at 1 day postvaccination. Principal component analysis (PCA) showed that the mRNA transcripts of innate immune genes in animals that received s.c. BNT162b2 clustered separately from those that received the vaccine via the i.m. route (S4A Fig); mRNA levels of these same genes from animals that received placebo clustered together despite the different routes of delivery (S4A Fig). These findings collectively suggest that the pro-inflammatory response is not a result of i.m. inoculation itself but of i.m. BNT162b2 vaccination. Volcano plots of the mRNA transcript levels found that, except for 2 genes, s.c. inoculation triggered lower expression of innate immune genes than those activated by i.m. vaccination (S4B Fig). Closer inspection of the pathways within the innate immune response showed significantly reduced expression of genes in the pro-inflammatory (Fig 6E, S4C–S4F Fig) and complement pathways (Fig 6F, S4G–S4J Fig). These findings indicate that s.c. inoculation activated significantly lower levels of pro-inflammatory responses that correlated with the lack of weight loss observed compared to animals that were vaccinated via the i.m. route [14]. In contrast, we observed comparable levels of expression in the genes in the interferon (IFN) pathway (Fig 6G, S4K–S4N Fig), which is known to be important for shaping the adaptive immune responses [25].

### Inoculation of BNT162b2 mRNA vaccine via the s.c. route does not compromise its immunogenicity

**Fig 7:**
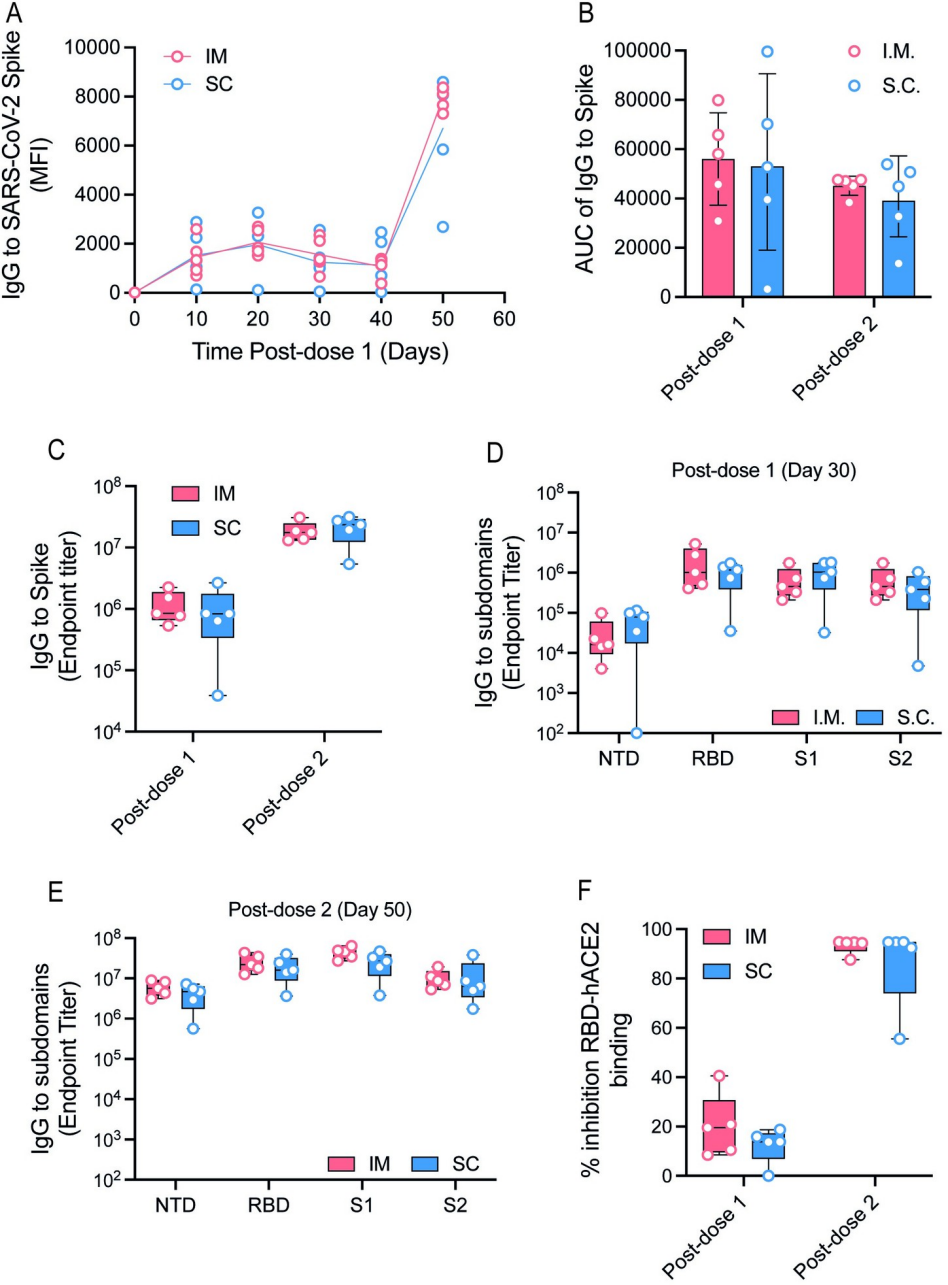
Vaccination induced antibody response was comparable in animals vaccinated via the s.c. as compared to i.m. routes. **(A, B)** IgG against the SARS-CoV-2 Spike protein over time (A) and total AUC (B), assessed with a whole spike protein in a Luminex immune-assay (readout as MFI). **(C**) IgG endpoint titers to SARS-CoV-2 whole spike 30 days postdose 1 and 10 days postdose 2. **(D, E)** IgG endpoint titer to SARS-CoV-2 NTD, S1, S2, and RBD proteins at 30 days postdose 1 (D) and 10 days postdose 2 (E). **(F**) Inhibition of RBD binding to hACE2 receptor 30 days postdose 1 and 10 days postdose 2 of vaccination represented as a percentage. Data presented as means ± SD. In vivo experiments were conducted with a minimum of *n* = 5 per group. Unpaired Student *t* test was used for experiments comparing 2 groups. *P < 0.05. Data underlying this figure can be found in S1 Data. AUC, area under the curve; i.m., intramuscular; MFI, mean fluorescence intensity; NTD, N-terminal domain; RBD, receptor binding domain; SARS-CoV-2, Severe Acute Respiratory Syndrome Coronavirus 2; s.c., subcutaneous.

To determine if indeed, as suggested by the lack of major differences in the induction of genes in the IFN pathway, we measured the ensuing antibody response in the original group of mice at 10, 20, 30, and 40 days post-first dose 1 and again at 10 days postdose 2 (S3 Fig). Longitudinal measurements of anti-S IgG levels showed no statistically significant difference in the antibody development kinetics following vaccination (Fig 7A), with comparable mean area under the curve (AUC) of anti-S IgG in the first 40 days after the first dose and 10 days after the second dose (Fig 7B). The endpoint titers of IgG that bound the N-terminal domain (NTD), receptor binding domain (RBD), the S1 and S2 subunits of the S protein were also comparable between s.c. and i.m. routes at the end of the 2-dose vaccination series (Fig 7C–7E). The percentage of inhibition of RBD binding to human ACE2, a surrogate of virus neutralization, was also comparable (Fig 7F).

**Fig 8:**
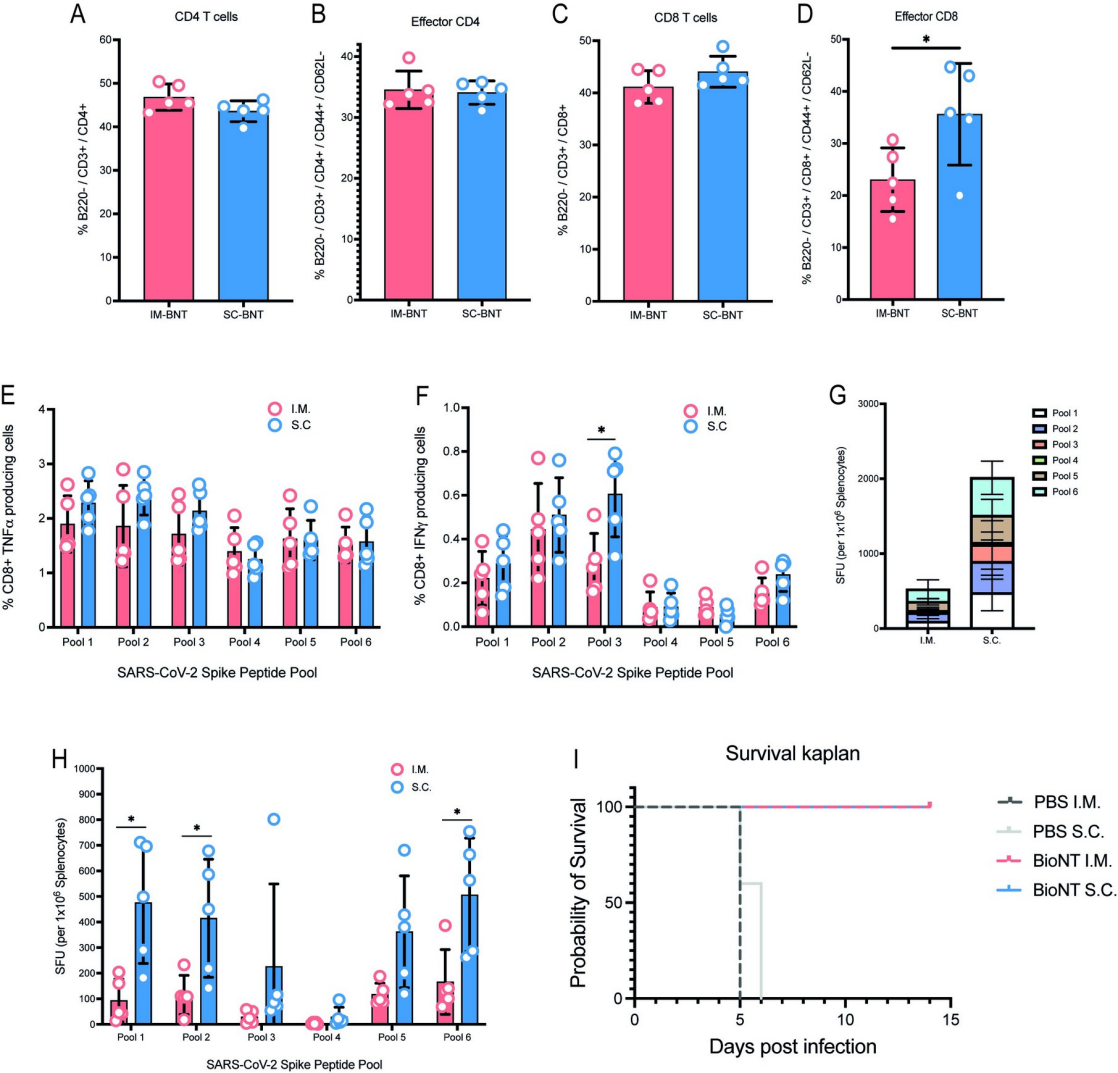
Superior T-cell responses generated after 2 doses of s.c. and i.m. BNT162b2 vaccination. **(A–D)** CD4^+^ (A), effector CD4^+^ (B), CD8^+^ (C) and effector CD8^+^ (D) cells were assessed in spleens of vaccinated animals with surface staining for T-cell markers via flow cytometry. **(E, F)** TNFα CD8^+^ T cells (E) and IFNγ^+^ CD8^+^ T cells (F) in spleens of immunized mice 10 days postdose 2 were assessed after ex vivo stimulation with pooled Spike protein peptides and subsequent intracellular staining. **(G, H)** SARS-CoV-2 spike protein-specific responses to pooled Spike protein peptides were assessed with IFNγ ELISpot assay 10 days postboost. **(I)** Kaplan–Meier survival curve of animal challenged with live SARS-CoV-2 12 days following second dose of vaccination. Data presented as means ± SD. In vivo experiments were conducted with a minimum of *n* = 5 per group. Unpaired Student *t* test was used for experiments comparing 2 groups. *P < 0.05. Data underlying this figure can be found in S1 Data. IFNγ, interferon gamma; i.m., intramuscular; SARS-CoV-2, Severe Acute Respiratory Syndrome Coronavirus 2; s.c., subcutaneous; TNFα, tumor necrosis factor alpha.

We next explored how s.c. inoculation affected T-cell response to BNT162b2 vaccination. We thus inoculated another group of mice and harvested the splenocytes at 10 days postvaccination. CD4 and CD8 T-cell effector and memory subsets were identified by surface expression of CD62L and CD44 (S5A–S5C Fig). Analysis of splenocytes showed comparable levels of CD4 and effector CD4 levels in both sets of mice (Fig 8A and 8B). For CD8 T cells, however, although the counts of total CD8 T cells were comparable (Fig 8C), there were significantly more effector CD8 T cells in animals that were vaccinated via the s.c. compared to the i.m. route (Fig 8D). Intracellular cytokine staining further revealed that while the proportion of CD8 T cells that produced tumor necrosis factor alpha (TNFα) was comparable (Fig 8E), the proportion that expressed IFNγ upon stimulation with pool 3 of the S protein was greater in animals vaccinated via the s.c. compared to i.m. route (Fig 8F). ELISpot analysis showed significantly higher number of S protein reactive T cells, especially in response to pools 1, 2, and 6, in animals vaccinated via the s.c. compared to i.m. route (Fig 8G and 8H). These findings collectively suggest vaccination using the s.c. route compared to the currently used i.m. route could potentially provide better cellular immunogenicity.

Finally, we examined if modifying the route of administration would impact the overall vaccine efficacy. K18-hACE2 mice were vaccinated with either BNT162b2 or PBS via the s.c. or i.m. route and challenged with live SARS-CoV-2 at day 12 postdose 2 of vaccination. All animals that received PBS via i.m. and all but 1 that received PBS via s.c. succumbed to SARS-CoV-2 infection by day 7. BNT162b2 vaccinated animals, via either s.c. or i.m. all survived (Fig 8I). Overall, our study suggest administration of the BNT162b2 vaccine can be delivered via the s.c. route to reduce reactogenicity without compromising vaccine efficacy.

## Discussion

Despite the remarkable efficacy of mRNA vaccines in protecting against COVID-19, there remains some level of concern about the reactogenicity of this vaccine that has contributed to vaccine hesitancy [9–12]. This has not only limited uptake of this 2-dose vaccine in some countries, but it could also limit the uptake of the third or booster dose, which has started for specific at-risk populations. Understanding the molecular underpinnings of AEs following immunization with mRNA vaccines would thus be important, not only for public health authorities to assuage safety concerns, but also to enable a less reactogenic approach to mRNA vaccination.

Our study revealed elevated baseline levels of genes enriched in T and NK cells in participants who developed moderately severe fatigue following BNT162b2 vaccination. Baseline expression of genes associated T and NK cell activity were positively correlated with expression of genes enriched in monocytes and neutrophils, as well as genes driving immune activation and TLR and inflammatory signaling. Among these immune activation and TLR and inflammatory signaling genes were IFNGR2, TLR8, TLR4, TLR1, and CSF2RB, which correlated with AEs following live-attenuated yellow fever virus vaccination [14]. These findings thus suggest that baseline expression of genes in T and NK activation may have functional roles and not just statistical association in increasing susceptibility to postvaccination fatigue.

While pathways associated with T and NK cell activities were enriched in those that developed moderately severe fatigue at baseline, a closer inspection of the differentially enriched genes suggested that these cells had an inhibitory phenotype. Specifically, we found increased baseline TIGIT and KLRF1 expression in participants with moderately severe fatigue; these genes were previously implicated in T-cell exhaustion [19,29,30]. Furthermore, TIGIT and another gene, KLRD1, both encode immunoreceptor tyrosine-based inhibitory motifs (ITIM)-containing receptors that are known to inhibit T and NK cell–mediated cytotoxicity, respectively [20–23]. Notably, several of the genes that we found enriched at baseline in those with moderately severe fatigue overlapped with those from studies on chronic fatigue syndrome (CFS). Studies have found that immune dysregulation, including altered T-cell metabolism [31,32], reduced NK cell cytotoxic and cytolytic activities [33,34] were associated with CFS. An obvious interpretation of our results would thus be that T and NK cell suppression or exhaustion predispose vaccinees to postvaccination fatigue. Interestingly, it was previously reported that T cells were crucial in tempering the early innate response, where a deficit of T cells resulted in higher abundance of pro-inflammatory cytokine upon antigen stimulation, in vivo [35]. It is thus possible that, in addition to T-cell abundance, T-cell suppression or exhaustion at baseline exacerbates innate immune response to vaccination and hence postvaccination fatigue.

There is another plausible explanation for our findings. We have sampled only the peripheral blood of our participants. The baseline gene expression in circulating T and NK cell may instead reflect a homeostatic response to activated T and NK cells resident in other tissues such as the lymph nodes. As vaccination would activate and expand adaptive immune cells in draining lymph nodes, baseline activated states of resident T and NK cells could predispose to overactivation of the innate immune response to result in fatigue. However, such studies will require invasive sampling procedures in sizeable number of volunteers that are not immediately feasible at this time. Future studies will be needed to determine which of these explanations are correct, as well as the cause of T and NK cell activation or exhaustion.

While we have identified baseline states of T and NK cell as correlated with increased susceptibility to postvaccination fatigue, the cause of such baseline states is unknown at this time. As with our previous study on the baseline correlates of symptomatic outcome from live-attenuated yellow fever vaccination, several factors, including host genetics and microbiome, could influence such states. We have also not experimentally shown a functional role for these cells in mediating this systemic AE. Indeed, it is possible that stimulating NK cells in mice, using interleukin (IL)-15 or a cocktail of cytokines, could test the hypothesis generated by our clinical study. However, substantial amount of optimization experiments would be required to reproduce the baseline gene expression profile of our vaccinees who developed postvaccination fatigue. Instead of pursuing this line of experiment, we turned our attention to asking if there was a simple solution for reducing systemic AEs without compromising BNT162b2 immunogenicity.

Inoculation of mRNA vaccines have been previously explored for biodistribution and immunogenicity, although not for reactogenicity, unlike those developed using conventional vaccine platforms [36,37]. Our mouse study thus adds to this body of knowledge by showing that s.c. inoculation of BNT162b2 could be more tolerable than the currently used i.m. inoculation. Reduced prevalence and extent of weight loss in our mouse model was reinforced by the finding of significantly fewer genes in the complement and pro-inflammatory pathways and at lower level of expression with s.c. compared to i.m inoculation. Importantly, immunogenicity was not compromised by the change in route of administration, which was consistent with the sustained expression of genes in the antiviral and IFN pathway.

Unexpectedly, our findings showed that cellular immune response with s.c. inoculation was superior to that of i.m. The reason for this increase in CD8 T-cell response is unclear to us at this time. Indeed, in our cohort of vaccinees, a statistically nonsignificant trend of lower T-cell response was seen in those with moderately severe fatigue compared to those without. However, our mouse study does suggest that inflammation may inhibit CD8 T-cell response. This suggestion is consistent with the finding that in severe COVID-19 patients, where hyperinflammation is a feature [38 39], T-cell response is muted compared to those with uncomplicated acute illness [40]. This finding could have translational implications as both experimental [41] and clinical [40,42–46] studies have consistently shown the importance of cellular immunity in protecting against COVID-19. Depletion of CD8 T cells but not CD19 B cells in immunized mice resulted in breakthrough SARS-CoV-2 infection in this same mouse model that we have used [41]. Early onset of T-cell response has also been shown to be associated with milder course of disease and rapid SARS-CoV-2 clearance in COVID-19 patients [40]. More recently, the presence of T cells that cross react with SARS-CoV-2 encoded proteins, such as the polymerase, was found to be capable of aborting symptomatic infection outcome [44,45]. Finally, the timing of S-reactive T cells from BNT162b2 vaccination has been shown to be associated with the onset of vaccine efficacy [47–49]. COVID-19 mRNA vaccination using the s.c. route may thus have added advantage of improved cellular immunogenicity.

## Conclusions

In conclusion, our findings suggest that elevated baseline levels of transcripts associated T and NK cell activity and function are susceptibility factors for postmRNA vaccination fatigue. Furthermore, s.c. inoculation of mRNA vaccines could be a pragmatic approach to reducing the rate and severity of systemic AEs without compromising vaccine efficacy.

## Materials and methods

### Clinical trial design

This study was approved by the SingHealth Centralized Institutional Review Board (CIRB/F 2021/2014). Healthcare workers from the Singapore Health Services institutions who were eligible for COVID-19 vaccination were invited to participate in this study, and written informed consent was obtained. Whole blood samples were collected for microarray profiling at D0 (predose1), D1, D20 (predose 2), and D21 and for T-cell response analysis at D0 (prevaccination), D7, D10, and D20 after vaccination with the Pfizer-BioNTech BNT162b2 vaccine.

### Microarray profiling of gene expression with microarray

Whole blood samples were collected in Tempus blood RNA tubes (Thermo Fisher Scientific, USA) from participants at D0 (predose 1), D1, D20 (predose 2), and D21 of vaccination with the Pfizer-BioNTech BNT162b2 vaccine. RNA isolation was performed according to the manufacturer’s protocol (Tempus Spin RNA Isolation Kit, Thermo Fisher Scientific). Microarray was performed with the Affymetrix Human Gene Chip 2.0 ST array at the Duke-NUS Genome Facility Genome Biology Core Facility. Data normalization was conducted using Partek Genomics Suite Analysis v.7 software. For GSEA at baseline, genes were preranked according to the log2 (FC) comparing participants with moderately severe fatigue to either participants with mild fatigue or no systemic AEs. GSEA was conducted using the previously established BTM [18]. Unsupervised hierarchical clustering was performed using Seaborn’s Clustermap function in Python version 3. Pearson correlation was conducted to correlate baseline gene expression with gene expression 1 day postvaccination. Genes were then ranked based on the correlation coefficient (r) and preranked GSEA using the BTM was performed.

### nCounter profiling of gene expression

RNA from whole blood was isolated from Tempus blood RNA tubes according to the manufacturer’s protocol (Tempus Spin RNA Isolation Kit, Thermo Fisher Scientific). Moreover, 50 ng of RNA was hybridized to reporter and capture probe sets of the nCounter Human Immunology v2 panel (Nanostring Technologies) at 65 °C for 24 hours. For in vivo experiments, 100 μL of whole blood collected 1 day postvaccination was lysed with BD PharmLyse reagent. RNA was subsequently extracted with the QIAGEN RNAeasy micro kit. 50ng of RNA was hybridized to the nCounter Mouse Inflammation v2 panel (NanoString Technologies) as previously described [28]. Unsupervised PCA performed to visualize variability between immunization routes was performed with Partek Genomics Suite Analysis v.7 software. Hierarchical clustering was performed with Seaborn’s Clustermap function in Python version 3.

### SARS-CoV-2–specific T-cell quantification

SARS-CoV-2–specific T cells were tested as described previously [47]. Briefly, thawed cryopreserved peripheral blood mononuclear cells (PBMCs) isolated from whole blood samples or freshly isolated splenocytes were subjected to IFN-γ ELISpot. Cells were stimulated overnight with either a 15 mer peptide library covering the entire SARS-CoV-2 spike protein divided into 6 pools at a final concentration of 1 μg/ml (for splenocytes) or a pool of 55 peptides covering the immunogenic regions of the SARS-CoV-2 S-protein (for PBMCs). Plates were subsequently washed and incubated with biotinylated IFNγ detection antibody, streptavidin-ALP and BCIP/NBT. Spots were imaged with ImmunoSpot Analyzer and quantified with ImmunoSpot software.

### Animal studies and study approvals

All animal studies were conducted in accordance with protocols approved by the IACUC at Singapore Health Services, Singapore (Ref no: 2020/SHS/1554). Female B6;SJL-Tg(K18-hACE2)2Prlmn/J (hACE2) mice were purchased from In Vivos Laboratory, Singapore. Residual reconstituted BNT162b2 that were to be disposed after daily vaccination of healthcare workers at the Singapore General Hospital were collected and stored at −80 °C until use. Vaccine used in each experiment were thawed, pooled to ensure homogeneity before being aliquoted into separate syringes for inoculation. Groups of 10- to 12-week-old wild-type hACE2 mice were vaccinated via the s.c. or i.m. route with 50 μL of BNT162b2 (Pfizer-BioNTech) for a final dosage of 5 μg. Submandibular bleeds were performed for serum isolation to assess SARS-CoV-2–specific humoral immune responses and transcriptomic studies. Ten days postprime and boost, mice were humanely killed, and spleens harvested for investigation of T-cell responses as previously reported. Briefly, spleens were passed through a 70 μm cells strainer (Corning, USA). Red blood cells were removed by lysis with BD PharmLyse reagent.

### Animal challenge study

SARS-CoV-2 challenge experiments were conducted with female B6;SJL-Tg(K18-hACE2)2Prlmn/J (hACE2) mice purchased from In Vivos Laboratory. Groups of 8- to 10-week-old wild-type female mice were vaccinated i.m. or s.c. with 50 μL of BNT162b2 (5 μg). Animals were infected with 5 × 10^4^ PFU in 50 μL via the intranasal route. Daily weight measurements and clinical scores were obtained. Mice were humanely killed when exhibiting >20% weight loss or clinical score of 10.

### Flow cytometry

Freshly isolated splenocytes were stained for the following antibodies: B220 (RA-6B2), CD3 (17A2), CD4 (RM4-5), CD8 (53–6.7), CD62L (MEL-14), and CD44 (IM7). Intracellular staining was performed by stimulating freshly isolated splenocytes with SARS-CoV-2 spike protein peptide pools for 6 hours at 37 °C. After stimulation, surface staining of CD3, CD4, and CD8 was performed, followed by intracellular staining of IFNγ (XMG1.2) and TNFα. Flow Cytometry was performed with the BD LSRFortessa Flow Cytometer and data analyzed with FlowJo.

### SARS-CoV-2–specific IgG Luminex immunoassay

Mouse serum IgG were measured as previously described [41]. Briefly, magpix beads were covalently conjugated with purified recombinant proteins (Ectodomain Spike, NTD, RBD, and S1 or S2). Beads were blocked with BSA, then probed for 1 hour at 37 °C with mouse serum diluted once at 1:100 (for MFI measurements) or in a dilution series (for estimation of IgG endpoint titers). Beads were then incubated with anti-human IgG-PE (Invitrogen, USA) for 30 minutes at 37°C, followed by data acquisition using a Magpix instrument. IgG endpoint titers were measured as previously described [41].

### sVNT assay

sVNT assay is an assay that assesses the inhibition of viral RBD binding to the human receptor, hACE2, and, therefore, is used as a proxy for virus neutralization [26]. We measured the inhibition of RBD binding to hACE2 using the commercial cPASS kit (Genscript, Singapore) as per manufacturer guidelines with a minor change in the serum dilution. Briefly, mouse sera were diluted 1:2,000 in sample dilution buffer, then incubated with diluted HRP conjugated RBD for 30 minutes at 37 °C. Mixture is then added to ELISA plates coated with hACE2 and incubated for 15 minutes at 37 °C.

### Statistical analysis

In vivo experiments were performed with either 3 or 5 animals per group. A 2-tailed unpaired Student *t* test was performed to compare the means of 2 conditions using Graphpad Prism (v9). For all datasets, a *P* value of less than 0.05 was considered significant. Numerical data used in all main figures are included in S1 Data, and data used in all Supporting information figures are included in S2 Data.

## Supporting information

S1 Fig Increased levels of genes in pathways associated with T and NK cell activity, categorized by fatigue severity, measured using the nCounter. **(A–C)** Mean log2 signal of all genes in the top enriched pathways, (A) enriched in T cells, (B) enriched in NK cells, and (C) T-cell activation at predose 1. **(D–F)** Mean log2 signal of all genes in the top enriched pathways, (D) enriched in T cells, (E) enriched in NK cells, and (F) T-cell activation at predose 2. **(G)** Gene expression of the top LEGs in the top enriched pathways at predose 1 and 2. Z scores of raw log2 counts are displayed. Data presented as means ± SD. Unpaired Student *t* test were used for experiments comparing 2 groups. *P < 0.05, **P < 0.01, ****P < 0.0001. Data underlying this figure can be found in S2 Data. LEG, leading edge gene.
(PDF)

S2 Fig Correlation of baseline T and NK cell genes with gene expression responses at 1 day postvaccination and immune response postvaccination. **(A, B)** Correlation for baseline expression of KLRF1 with enriched in monocytes gene sets (A) and cell cycle and transcription gene sets (B) at 1 day postdose 1. **(C, D)** Heatmap of significantly increased genes in the moderately severe fatigue group of pathways positively correlated with levels of T and NK cell genes at predose 1 (C) and predose 2 (D). Mean z-scores of raw log2 FC (day 1 postvaccination/predose) are displayed. **(E)** Inhibition of RBD binding to hACE2 receptor measured using the commercial cPASS kit at 1:20 serum dilution. **(F)** SARS-CoV-2 spike protein-specific responses to pooled Spike protein peptides were assessed with IFNγ ELISpot assay at days 0, 7, 10, and 21 of first dose of vaccination. Data underlying this figure can be found in S2 Data. FC, fold change; IFNγ, interferon gamma; KLRF1, killer cell lectin-like receptor F1; RBD, receptor binding domain; SARS-CoV-2, Severe Acute Respiratory Syndrome Coronavirus 2.
(PDF)

S3 Fig Study design of in vivo experiments and in vivo clinical scoring sheet. Summary schematic of the 3 animal studies performed. In Study 1, hACE2 mice (*n* = 5/group) were immunized with 5 μg of BNT162b either by the s.c. or i.m. route at day 0 and 40. Blood drawn 10, 20, 30, and 40 days post-first dose and subsequently 10 days post-second dose of vaccination was used for antibody quantification. Ten days post-second dose, splenocytes harvested were analyzed for cellular T-cell responses by flow cytometry and ELISpot. Weights and clinical scores were monitored for 10 days postvaccination. In Study 2, hACE2 mice (*n* = 3/group) were immunized with either PBS or 5 μg of BNT162b either by the s.c. or i.m. route. Blood was drawn 1 day postvaccination for whole blood transcriptomics. In Study 3, hACE2 mice (*n* = 5/group) were immunized with 5μg of BNT162b either by the s.c. or i.m. route. Ten days post-first dose, splenocytes harvested were analyzed for cellular T-cell responses by ELISpot. i.m., intramuscular; s.c., subcutaneous.
(PDF)

S4 Fig S.c. and i.m. BNT162b vaccination resulted on upregulated inflammatory and immune genes. **(A)** PCA of inflammatory and immune gene expression following vaccination with PBS or BNT162b via the s.c. or i.m. route. **(B)** Volcano plots of s.c. versus i.m. BNT162b vaccinated gene FCs (x-axis) and log_10_ *P* value (y-axis). (**C–F)** Inflammatory pathway genes TNFα (C), IL6 (D), MAP2K1 (E), and NOS2 (F) normalized to their respective PBS controls expressed in whole blood of s.c. and i.m. BNT162b vaccinated K18-hACE2 animals. **(G–J)** Complement pathway genes C1RA (G), C2 (H), C7 (I), and CFD (J) normalized to their respective PBS controls expressed in whole blood of s.c. and i.m. BNT162b vaccinated K18-hACE2 animals. **(K–N)** IFN pathway genes IFNα (K), TLR7 (L), IRF7 (M), and IFIT1 (N) normalized to their respective PBS controls expressed in whole blood of s.c. and i.m. BNT162b vaccinated K18-hACE2 animals. Data presented as means ± SD. Unpaired Student *t* test were used for experiments comparing 2 groups. *P < 0.05, **P < 0.01, ****P < 0.0001. Data underlying this figure can be found in S2 Data. FC, fold change; IFN, interferon; i.m., intramuscular; PCA, principal component analysis; s.c., subcutaneous; TLR, Toll-like receptor; TNFα, tumor necrosis factor alpha.
(PDF)

S5 Fig Schematic of SARS-CoV-2 spike protein peptide pools and representative gating strategy for flow cytometry. **(A)** Summary schematic of the 6 peptide pools of the SARS-CoV-2 spike protein used for stimulating T cells in mouse splenocytes. **(B)** Representative gating strategy illustrating splenocyte population being subgated to CD44 and CD62L expressing CD4^+^ and CD8^+^ cells. **(C)** Representative gating strategy illustrating splenocyte population being subgated to TNFα, IL4, IL2, and IFNγ expressing CD4^+^ and CD8^+^ cells. IFNγ, interferon gamma; IL, interleukin; SARS-CoV-2, Severe Acute Respiratory Syndrome Coronavirus 2; TNFα, tumor necrosis factor alpha.
(PDF)

S1 Table Participant demographics. A total of 32 participants from the healthcare workers cohort were selected for analysis. Moreover, 5 moderately severe fatigue and 11 mild fatigue cases were age- and gender- matched with participants with no reported AE. AE, adverse event.
(PDF)

S2 Table. Guidelines for grading of fatigue. Participants were monitored for onset of AEs within the first 7 days of receiving dose 1 and dose 2 of the vaccine. Participants who reported AEs were followed up until resolution of AEs. The grading system for fatigue follows the guidelines set by the US FDA on preventive vaccine clinical trials. AE, adverse event; FDA, Food and Drug Administration (PDF)

S3 Table Clinical monitoring sheet for in vivo study. Clinical symptoms tracked in animals vaccinated with BNT162b for the first 10 days postvaccination. Any animal with a score of 5 in a single category, overall score of 10 or weight loss of more than 20% is euthanized. Clinical monitoring sheet is adapted from the University of British Columbia Animal Care and Use program.
(PDF)

S1 Data Excel spreadsheet containing, in separate sheets, all the numerical data underlying Figs 2A–2D, 2F, 3A–3D, 4A–4F, 5A–5F, 6A–6D, 7A–7F, and 8A–8I. (XLSX)

S2 Data Excel spreadsheet containing, in separate sheets, all the numerical data underlying S1A–S1F, S2A, S2B, S2E, S2F, and S4B–S4N Figs. (XLSX)

## Abbreviations

AE: adverse event
AUC: area under the curve
BTM: blood transcription module
CFS: chronic fatigue syndrome
COVID-19: Coronavirus Disease 2019
CTCAE: Common Terminology Criteria for Adverse Events
FC: fold change
GSEA: gene set enrichment analysis
i.m.: intramuscular
IFN: interferon
IFNγ: interferon gamma
IL: interleukin
ITIM: immunoreceptor tyrosine-based inhibitory motif
K18-hACE2: K18 human angiotensin converting enzyme 2
KLRF1: killer cell lectin-like receptor F1
LEG: leading edge gene
NTD: N-terminal domain
PCA: principal component analysis
PMBC: peripheral blood mononuclear cell
RBD: receptor binding domain
s.c.: subcutaneous
SARS-CoV-2: Severe Acute Respiratory Syndrome Coronavirus 2
sVNT: surrogate virus neutralization test
TIGIT: T-cell immunoglobulin and ITIM domain
TLR: Toll-like receptor
TNFα: tumor necrosis factor alpha
