# Supplementary material for: Adverse effects following anti–COVID-19 vaccination with mRNA-based BNT162b2 are alleviated by altering the route of administration and correlate with baseline enrichment of T and NK cell genes
Source: PLoS Biol. 2022 May 31;20(5):e3001643. doi: 10.1371/journal.pbio.3001643 (PMC9154185; doi:10.1371/journal.pbio.3001643)
Supplement: S1 Fig — (A–C) Mean log2 signal of all genes in the top enriched pathways, (A) enriched in T cells, (B) enriched in NK cells, and (C) T-cell activation at predose 1. (D–F) Mean log2 signal of all genes in the top enriched pathways, (D) enriched in T cells, (E) enriched in NK cells, and (F) T-cell activation at predose 2. (G) Gene expression of the top LEGs in the top enriched pathways at predose 1 and 2. Z scores of raw log2 counts are displayed. Data presented as means ± SD. Unpaired Student t test were used for experiments comparing 2 groups. *P < 0.05, **P < 0.01, ****P < 0.0001. Data underlying this figure can be found in S2 Data. LEG, leading edge gene. (PDF) [file pbio.3001643.s001.pdf]

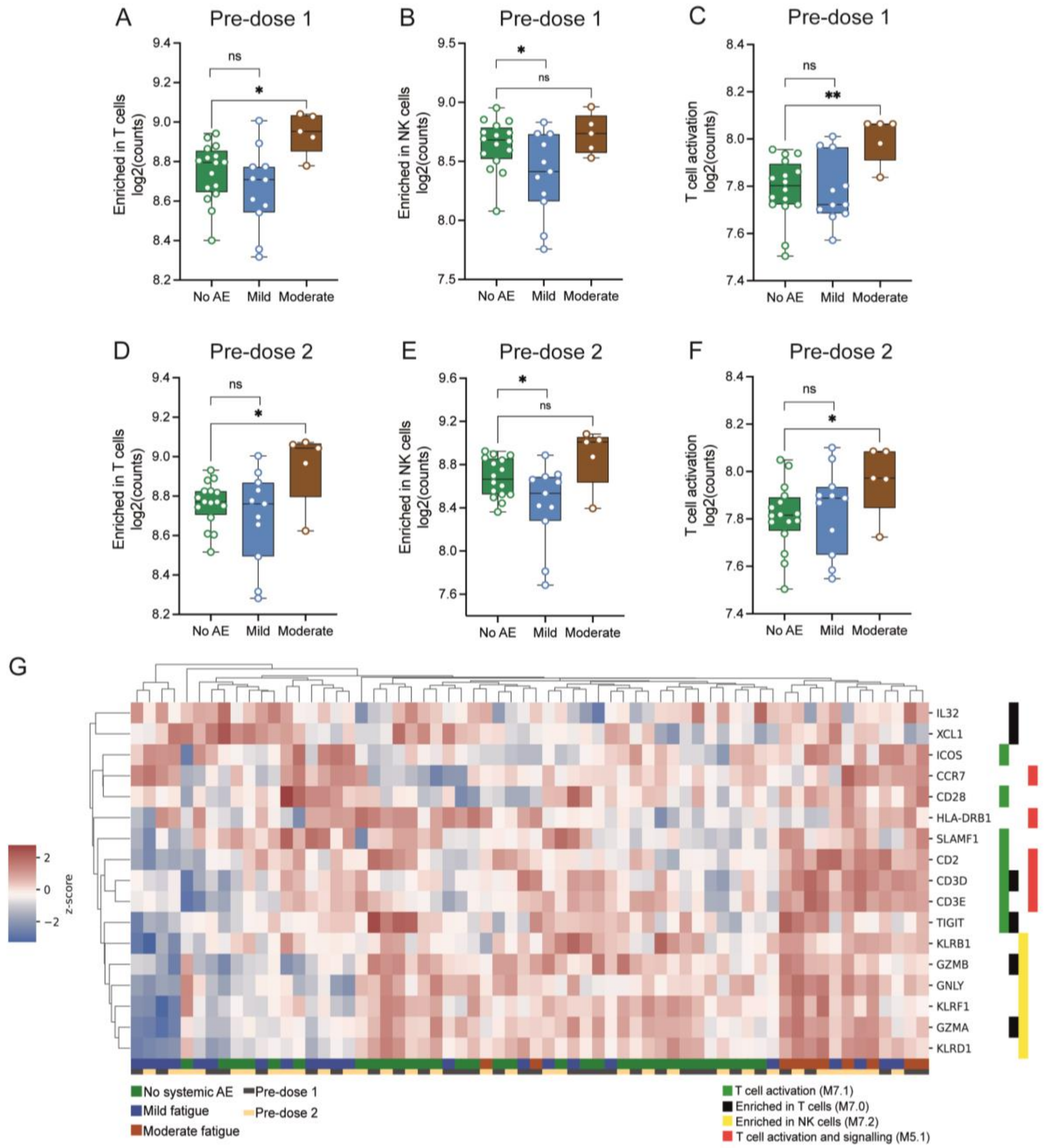

S1 Fig. Increased levels of genes in pathways associated with T and NK cell activity, categorized by fatigue severity, measured using the nCounter.
(A-C) Mean log2 signal of all genes in the top enriched pathways, (A) enriched in T cells, (B) enriched in NK cells, and (C) T cell activation at pre-dose 1.
(D-F) Mean log2 signal of all genes in the top enriched pathways, (D) enriched in T cells, (E) enriched in NK cells, and (F) T cell activation at pre-dose 2.
(G) Gene expression of the top leading edge genes in the top enriched pathways at pre-dose 1 and 2. Z scores of raw log2 counts are displayed.
*Data presented as means ± SD. Unpaired student's T test were used for experiments comparing 2 groups. *P < 0.05, **P < 0.01, ****P<0.0001.*
Data underlying this Figure can be found in S2_Data.
