# Supplementary material for: Adverse effects following anti–COVID-19 vaccination with mRNA-based BNT162b2 are alleviated by altering the route of administration and correlate with baseline enrichment of T and NK cell genes
Source: PLoS Biol. 2022 May 31;20(5):e3001643. doi: 10.1371/journal.pbio.3001643 (PMC9154185; doi:10.1371/journal.pbio.3001643)
Supplement: S2 Fig — (A, B) Correlation for baseline expression of KLRF1 with enriched in monocytes gene sets (A) and cell cycle and transcription gene sets (B) at 1 day postdose 1. (C, D) Heatmap of significantly increased genes in the moderately severe fatigue group of pathways positively correlated with levels of T and NK cell genes at predose 1 (C) and predose 2 (D). Mean z-scores of raw log2 FC (day 1 postvaccination/predose) are displayed. (E) Inhibition of RBD binding to hACE2 receptor measured using the commercial cPASS kit at 1:20 serum dilution. (F) SARS-CoV-2 spike protein-specific responses to pooled Spike protein peptides were assessed with IFNγ ELISpot assay at days 0, 7, 10, and 21 of first dose of vaccination. Data underlying this figure can be found in S2 Data. FC, fold change; IFNγ, interferon gamma; KLRF1, killer cell lectin-like receptor F1; RBD, receptor binding domain; SARS-CoV-2, Severe Acute Respiratory Syndrome Coronavirus 2. (PDF) [file pbio.3001643.s002.pdf]

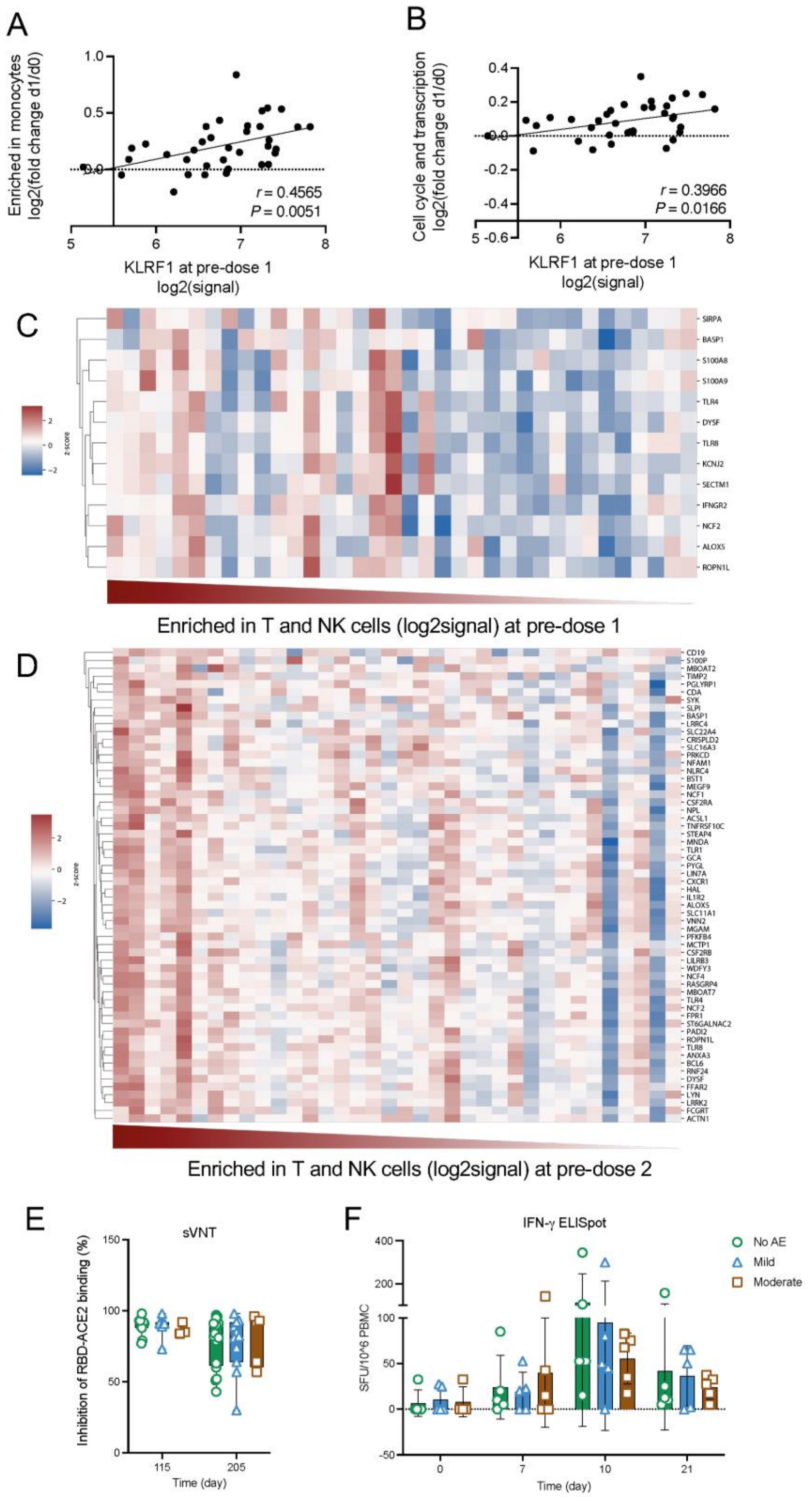

S2 Fig. Correlation of baseline T and NK cell genes with gene expression responses at one day post vaccination and immune response post vaccination.

(A-B) Correlation for baseline expression of KLRF1 with enriched in monocytes gene sets (A) and cell cycle and transcription gene sets (B) at one day post-dose 1.

(C-D) Heatmap of significantly increased genes in the moderately severe fatigue group of pathways positively correlated with levels of T and NK cell genes at pre-dose 1 (C) and pre-dose 2 (D). Mean z-scores of raw log2 FC (day 1 post-vaccination / pre-dose) are displayed.
(E) Inhibition of RBD binding to hACE2 receptor measured using the commercial cPASS kit at 1:20 serum dilution.
(F) SARS-CoV-2 spike protein-specific responses to pooled Spike protein peptides were assessed with IFNγ ELISpot assay at days 0, 7, 10 and 21 of first dose of vaccination.
Data underlying this Figure can be found in S2_Data.
