# Supplementary material for: Adverse effects following anti–COVID-19 vaccination with mRNA-based BNT162b2 are alleviated by altering the route of administration and correlate with baseline enrichment of T and NK cell genes
Source: PLoS Biol. 2022 May 31;20(5):e3001643. doi: 10.1371/journal.pbio.3001643 (PMC9154185; doi:10.1371/journal.pbio.3001643)
Supplement: S3 Fig — Summary schematic of the 3 animal studies performed. In Study 1, hACE2 mice (n = 5/group) were immunized with 5 μg of BNT162b either by the s.c. or i.m. route at day 0 and 40. Blood drawn 10, 20, 30, and 40 days post-first dose and subsequently 10 days post-second dose of vaccination was used for antibody quantification. Ten days post-second dose, splenocytes harvested were analyzed for cellular T-cell responses by flow cytometry and ELISpot. Weights and clinical scores were monitored for 10 days postvaccination. In Study 2, hACE2 mice (n = 3/group) were immunized with either PBS or 5 μg of BNT162b either by the s.c. or i.m. route. Blood was drawn 1 day postvaccination for whole blood transcriptomics. In Study 3, hACE2 mice (n = 5/group) were immunized with 5μg of BNT162b either by the s.c. or i.m. route. Ten days post-first dose, splenocytes harvested were analyzed for cellular T-cell responses by ELISpot. i.m., intramuscular; s.c., subcutaneous. (PDF) [file pbio.3001643.s003.pdf]

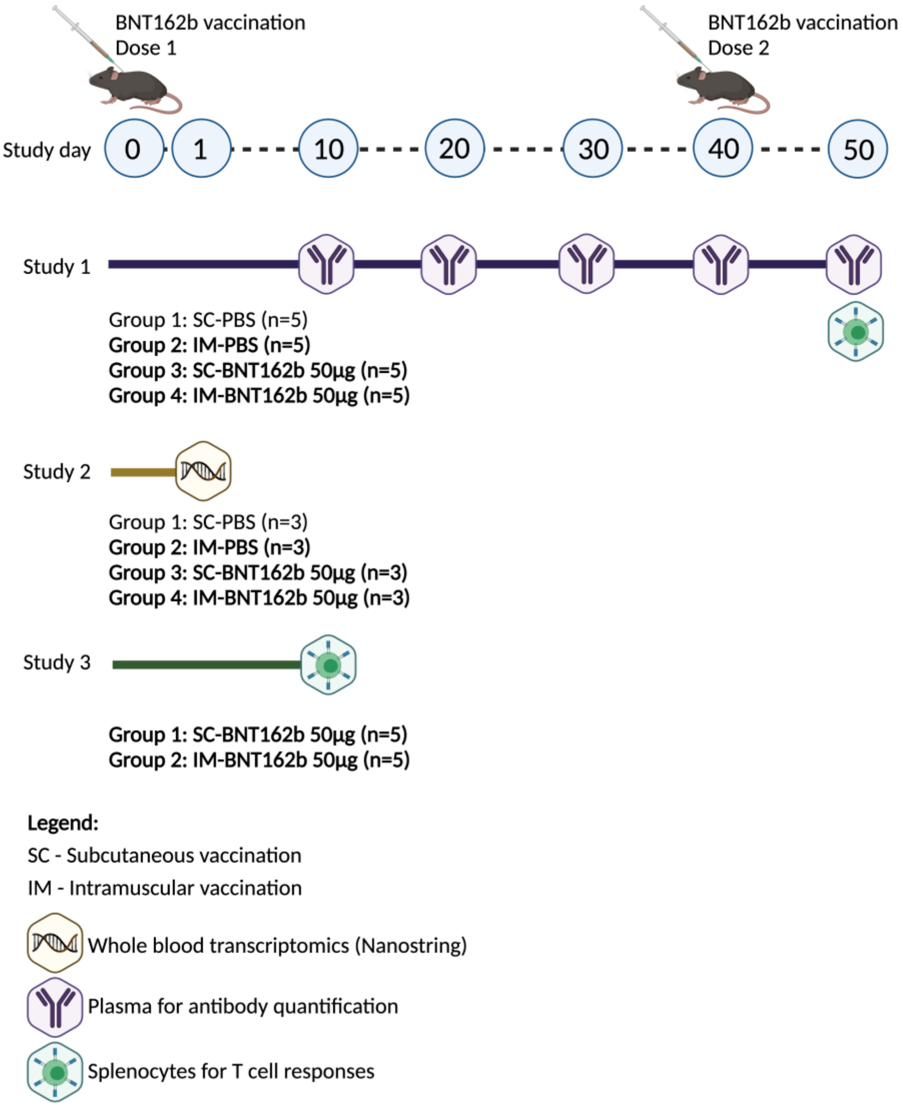

S3 Fig. Study design of *in vivo* experiments and *in vivo* clinical scoring sheet.
Summary schematic of the three animal studies performed. In Study 1, hACE2 mice (n=5/group) were immunized with 5µg of BNT162b either by the SC or IM route at day 0 and 40. Blood drawn 10, 20, 30 and 40 days post first dose and subsequently 10 days post second dose of vaccination was used for antibody quantification. 10 days post second dose, splenocytes harvested were analyzed for cellular T cell responses by flow cytometry and ELISpot. Weights and clinical scores were monitored for 10 days post vaccination. In Study 2, hACE2 mice (n=3/group) were immunized with either PBS or 5µg of BNT162b either by the subcutaneous (SC) or intramuscular (IM) route. Blood was drawn one day post-vaccination for whole blood transcriptomics. In Study 3, hACE2 mice (n=5/group) were immunized with 5µg of BNT162b either by the SC or IM route. 10 days post first dose, splenocytes harvested were analyzed for cellular T cell responses by ELISpot.
