# Supplementary material for: Adverse effects following anti–COVID-19 vaccination with mRNA-based BNT162b2 are alleviated by altering the route of administration and correlate with baseline enrichment of T and NK cell genes
Source: PLoS Biol. 2022 May 31;20(5):e3001643. doi: 10.1371/journal.pbio.3001643 (PMC9154185; doi:10.1371/journal.pbio.3001643)
Supplement: S4 Fig — (A) PCA of inflammatory and immune gene expression following vaccination with PBS or BNT162b via the s.c. or i.m. route. (B) Volcano plots of s.c. versus i.m. BNT162b vaccinated gene FCs (x-axis) and log10 P value (y-axis). (C–F) Inflammatory pathway genes TNFα (C), IL6 (D), MAP2K1 (E), and NOS2 (F) normalized to their respective PBS controls expressed in whole blood of s.c. and i.m. BNT162b vaccinated K18-hACE2 animals. (G–J) Complement pathway genes C1RA (G), C2 (H), C7 (I), and CFD (J) normalized to their respective PBS controls expressed in whole blood of s.c. and i.m. BNT162b vaccinated K18-hACE2 animals. (K–N) IFN pathway genes IFNα (K), TLR7 (L), IRF7 (M), and IFIT1 (N) normalized to their respective PBS controls expressed in whole blood of s.c. and i.m. BNT162b vaccinated K18-hACE2 animals. Data presented as means ± SD. Unpaired Student t test were used for experiments comparing 2 groups. *P < 0.05, **P < 0.01, ****P < 0.0001. Data underlying this figure can be found in S2 Data. FC, fold change; IFN, interferon; i.m., intramuscular; PCA, principal component analysis; s.c., subcutaneous; TLR, Toll-like receptor; TNFα, tumor necrosis factor alpha. (PDF) [file pbio.3001643.s004.pdf]

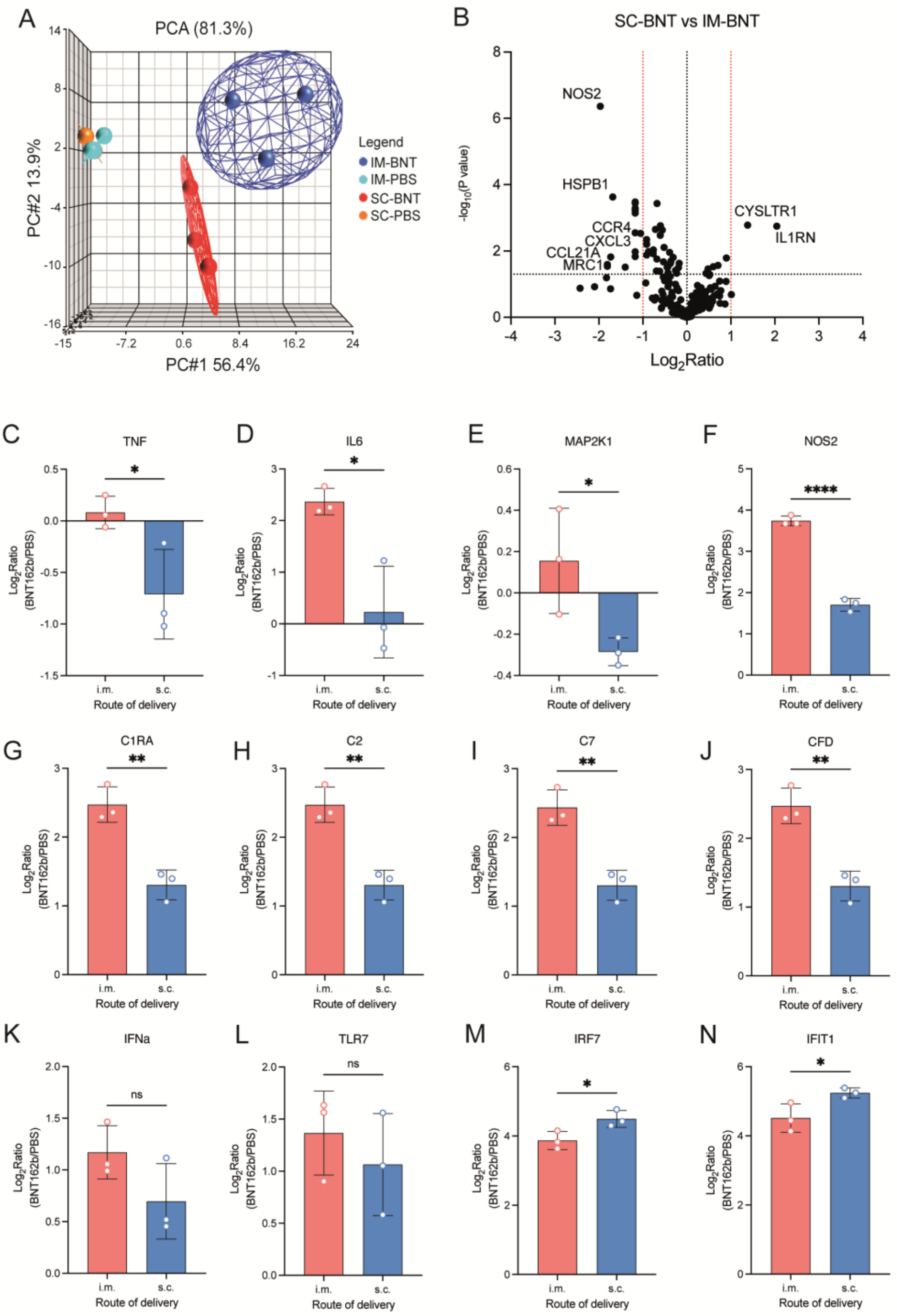

S4 Fig. Subcutaneous and intramuscular BNT162b vaccination resulted on upregulated inflammatory and immune genes.

(A) Principle-component analysis (PCA) of inflammatory and immune gene expression following vaccination with PBS or BNT162b via the subcutaneous (SC) or intramuscular (IM) route.

(B) Volcano plots of SC versus IM BNT162b vaccinated gene fold changes (x-axis) and $log_{10}p$ value (y-axis).

(**C-F)** Inflammatory pathway genes TNFα (C), IL6 (D), MAP2K1 (E) and NOS2 (F) normalized to their respective PBS controls expressed in whole blood of s.c. and i.m. BNT162b vaccinated K18-hACE2 animals.

(G-J) Complement pathway genes C1RA (G), C2 (H), C7 (I) and CFD (J) normalized to their respective PBS controls expressed in whole blood of s.c. and i.m. BNT162b vaccinated K18-hACE2 animals.

(K-N) Interferon pathway genes IFNα (K), TLR7 (L), IRF7 (M) and IFIT1 (N) normalized to their respective PBS controls expressed in whole blood of s.c. and i.m. BNT162b vaccinated K18-hACE2 animals.

*Data presented as means ± SD. Unpaired student's T test were used for experiments comparing 2 groups. *P < 0.05, **P < 0.01, ****P<0.0001*

Data underlying this Figure can be found in S2_Data.
