# Supplementary material for: Adverse effects following anti–COVID-19 vaccination with mRNA-based BNT162b2 are alleviated by altering the route of administration and correlate with baseline enrichment of T and NK cell genes
Source: PLoS Biol. 2022 May 31;20(5):e3001643. doi: 10.1371/journal.pbio.3001643 (PMC9154185; doi:10.1371/journal.pbio.3001643)
Supplement: S5 Fig — (A) Summary schematic of the 6 peptide pools of the SARS-CoV-2 spike protein used for stimulating T cells in mouse splenocytes. (B) Representative gating strategy illustrating splenocyte population being subgated to CD44 and CD62L expressing CD4+ and CD8+ cells. (C) Representative gating strategy illustrating splenocyte population being subgated to TNFα, IL4, IL2, and IFNγ expressing CD4+ and CD8+ cells. IFNγ, interferon gamma; IL, interleukin; SARS-CoV-2, Severe Acute Respiratory Syndrome Coronavirus 2; TNFα, tumor necrosis factor alpha. (PDF) [file pbio.3001643.s005.pdf]

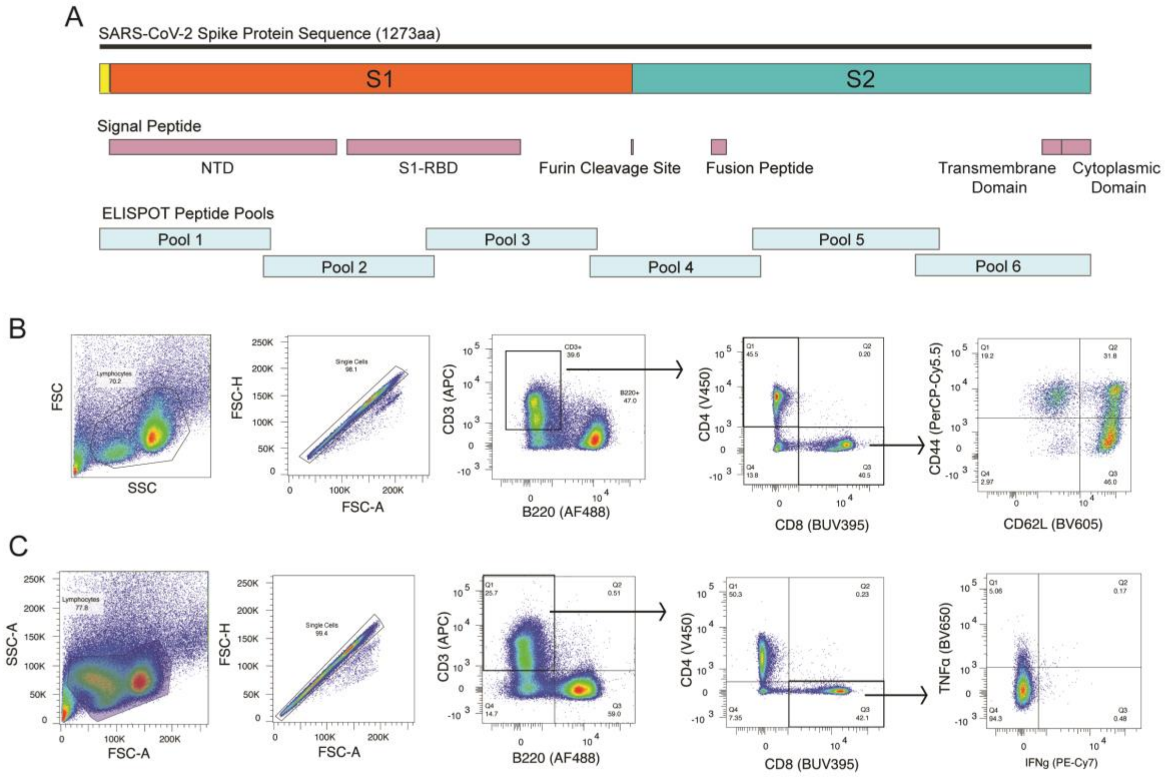

S5 Fig. Schematic of SARS-CoV-2 spike protein peptide pools and representative gating strategy for flow cytometry.

(A) Summary schematic of the 6 peptide pools of the SARS-CoV-2 spike protein used for stimulating T cells in mouse splenocytes.

(B) Representative gating strategy illustrating splenocyte population being subgated to CD44 and CD62L expressing $CD4^+$ and $CD8^+$ cells.

(C) Representative gating strategy illustrating splenocyte population being subgated to TNFα, IL4, IL2 and IFNγ expressing $CD4^+$ and $CD8^+$ cells.
