# Supplementary material for: Adverse effects following anti–COVID-19 vaccination with mRNA-based BNT162b2 are alleviated by altering the route of administration and correlate with baseline enrichment of T and NK cell genes
Source: PLoS Biol. 2022 May 31;20(5):e3001643. doi: 10.1371/journal.pbio.3001643 (PMC9154185; doi:10.1371/journal.pbio.3001643)
Supplement: S1 Table — A total of 32 participants from the healthcare workers cohort were selected for analysis. Moreover, 5 moderately severe fatigue and 11 mild fatigue cases were age- and gender- matched with participants with no reported AE. AE, adverse event. (PDF) [file pbio.3001643.s006.pdf]

S1 Table. Subject demographics. 32 subjects from the healthcare workers cohort were selected for analysis. 5 moderately severe fatigue and 11 mild fatigue cases were age- and gender- matched with subjects with no reported AE.

Subject Number	AE	Age	Gender	Ethnicity	Allergy	History of SARS-CoV-2 infection	Medical history	Height (m)	Weight (kg)	BMI
S128	Moderate Fatigue	34	Male	Chinese	NSAIDs	No	NIL	1.7	65.6	22.7
G006	Moderate Fatigue	25	Female	Chinese	No	No	NIL	1.68	50	17.72
G020	Moderate Fatigue	40	Male	Indian	No	No	Asthma	1.69	70	24.51
G024	Moderate Fatigue	30	Female	Chinese	No	No	NIL	1.69	59	20.66
G005	Moderate Fatigue	41	Male	Chinese	No	No	NIL	1.75	66	21.55
S025	Mild Fatigue	28	Female	Chinese	No	No	Gastritis, irritable Bowel Syndrome	1.67	80	28.69
S078	Mild Fatigue	50	Male	Chinese	no	No	High blood pressure, gout	1.69	93	32.56
S018	Mild Fatigue	32	Male	Chinese	No	No	NIL	1.83	81	24.19
S019	Mild Fatigue	39	Female	Chinese	No	No	Hypothyroidism	1.6	70	27.34
G001	Mild Fatigue	38	Male	Chinese	No	No	NIL	1.7	70	24.22
G003	Mild Fatigue	32	Female	Chinese	No	No	NIL	1.53	60	25.63
G016	Mild Fatigue	55	Male	Chinese	No	No	Hypertension	1.65	50	18.37
G019	Mild Fatigue	51	Male	Chinese	No	No	Asthma	1.8	80	24.69
S077	Mild Fatigue	53	Female	Chinese	no	No	NIL	1.72	65	21.97
S094	Mild Fatigue	33	Male	Chinese	No	No	NIL	1.66	105	38.1
G008	Mild Fatigue	41	Female	Chinese	No	No	NIL	1.66	54	19.6
G009	No	39	Female	Chinese	No	No	NIL	1.55	53	22.06
G023	No	47	Male	Chinese	No	No	NIL	1.72	68	22.99
G028	No	43	Male	Chinese	No	No	Fractured calcaneum s/p surgery	1.67	67	24.02
G031	No	54	Male	Chinese	No	No	Hypertension	1.7	58	20.07
S110	No	32	Female	Chinese	No	No	Left Hemithyroidectomy	1.61	47	18.13
S119	No	32	Male	Chinese	No	No	NIL	1.78	76	23.99
S008	No	45	Male	Filipino	No	No	NIL	1.7	71	24.57

S009	No	32	Male	Malay	No	No	NIL	1.7	67	23.18
S029	No	34	Male	Chinese	No	No	NIL	1.74	80	26.42
S041	No	53	Male	Chinese	no	No	NIL	1.6	65	25.39
S013	No	50	Female	Chinese	Chlormezanone	No	NIL	1.54	57	24.03
S060	No	27	Female	Chinese	no	No	Lasik	1.54	54	22.77
S070	No	44	Female	Chinese	no	No	Ovarian cystectomy	1.56	53	21.78
S081	No	31	Male	Chinese	sulfanamide	No	Appendectomy	1.76	64	20.66
S072	No	33	Female	Chinese	no	No	NIL	1.5	46	20.44
S073	No	29	Female	Chinese	no	No	Left level cervical lymph node incisional biopsy	1.63	64.25	24.18
