# Supplementary material for: Adverse effects following anti–COVID-19 vaccination with mRNA-based BNT162b2 are alleviated by altering the route of administration and correlate with baseline enrichment of T and NK cell genes
Source: PLoS Biol. 2022 May 31;20(5):e3001643. doi: 10.1371/journal.pbio.3001643 (PMC9154185; doi:10.1371/journal.pbio.3001643)
Supplement: S2 Table. Guidelines for grading of fatigue. Participants were monitored for onset of AEs within the first 7 days of receiving dose 1 and dose 2 of the vaccine. Participants who reported AEs were followed up until resolution of AEs. The grading system for fatigue follows the guidelines set by the US — (PDF) [file pbio.3001643.s007.pdf]

S2 Table. Guidelines for grading of fatigue. Subjects were monitored for onset of AEs within the first 7 days of receiving dose 1 and dose 2 of the vaccine. Subject who reported AEs were followed up until resolution of AEs. The grading system for fatigue follows the guidelines set by the US FDA on preventive vaccine clinical trials.

	**Mild (Grade 1)**	**Moderate (Grade 2)**	**Severe (Grade 3)**	**Potentially Life Threatening (Grade 4)**
Fatigue	No interference with activity	Some interference with activity	Significant; prevents daily activity	ER visit or hospitalization
