# Supplementary material for: Adverse effects following anti–COVID-19 vaccination with mRNA-based BNT162b2 are alleviated by altering the route of administration and correlate with baseline enrichment of T and NK cell genes
Source: PLoS Biol. 2022 May 31;20(5):e3001643. doi: 10.1371/journal.pbio.3001643 (PMC9154185; doi:10.1371/journal.pbio.3001643)
Supplement: S3 Table — Clinical symptoms tracked in animals vaccinated with BNT162b for the first 10 days postvaccination. Any animal with a score of 5 in a single category, overall score of 10 or weight loss of more than 20% is euthanized. Clinical monitoring sheet is adapted from the University of British Columbia Animal Care and Use program. (PDF) [file pbio.3001643.s008.pdf]

S3 Table. Clinical monitoring sheet for *in vivo* study. Clinical symptoms tracked in animals vaccinated with BNT162b for the first 10 days post vaccination. Any animal with a score of 5 in a single category, overall score of 10 or weight loss of more than 20% is euthanized. Clinical monitoring sheet is adapted from the Univeristy of British Columbia Animal Care and Use program.

Clinical Symptoms	Clinical Score
Nothing Abnormal	0
**Body Weight (g)**	
5-9% loss	1
10-14% loss	2
15-20% loss	3
**Hydration**	
Dry Eyes	1
Sunken Eyes	2
Signs of Weakness	
Mild Ruffled fur	1
Marked Ruffled fur	2
Hunched (while sitting)	2
Hunched (sit and walk)	3
**CNS Dysfunction**	
Abnormal Gait	1
Shaking (occasionally)	2
Shaking (constantly)	5
Paralysis	5
**Respiratory Distress**	
Nasal Discharge	1
Shallow Rapid Breathing	2
Abdomnal effort in breathing	3
Slow labored breathing	4
Gasping	4
**Lethargy**	
Slow moving	2
Slow when stimulated	3
No response when stimulated	5
